# Repurposing Rilmenidine as a Potential Antimetastatic Therapy Targeting Nischarin in Pancreatic Ductal Adenocarcinoma

**DOI:** 10.3390/cells15111032

**Published:** 2026-06-03

**Authors:** Kristina Živić, Marijana Pavlović, Marija Ostojić, Danijel Galun, Aleksandar Pavić, Tatjana Srdić-Rajić, Jelena Grahovac

**Affiliations:** 1 Department of Experimental Oncology, Institute for Oncology and Radiology of Serbia, 11000 Belgrade, Serbia; 2 School of Medicine, University of Belgrade, 11000 Belgrade, Serbia; 3 HPB Unit, Clinic for Digestive Surgery, University Clinical Center of Serbia, 11000 Belgrade, Serbia; 4 Institute of Molecular Genetics and Genetic Engineering, University of Belgrade, 11000 Belgrade, Serbia

**Keywords:** pancreatic cancer, nischarin, rilmenidine, drug repurposing, metastasis

## Abstract

Pancreatic ductal adenocarcinoma (PDAC) is one of the most aggressive cancer types with a dismal prognosis, where early metastatic dissemination and rich desmoplastic stroma limit the therapeutic efficacy. Nischarin (NISCH)/Imidazoline-1 receptor (IR-1) is a potential tumor suppressor that is involved in the regulation of cell migration, invasion, and cytoskeletal organization. Importantly, several clinically approved agonists have been shown to target this receptor. This study aimed to examine NISCH expression in PDAC and the effects of its agonists with the intent of drug repurposing. NISCH was expressed in PDAC tumor tissue, in both the epithelial and stromal compartments of tumors, and higher NISCH expression was associated with longer patient survival. Out of the three tested NISCH agonists, moxonidine, clonidine and rilmenidine, rilmenidine was the only one affecting cancer cell viability and at high doses induced cancer cell apoptosis. Transcriptome analysis of rilmenidine-treated PDAC cells revealed changes associated with cytoskeletal organization, translating into decreased adhesion and migration in vitro. In cancer-associated fibroblasts (CAFs), rilmenidine treatment decreased the expression of activation markers and limited cancer cell-CAF cytokine communication in the co-culture. Ultimately, in the in vivo tumor xenograft zebrafish model, rilmenidine reduced the metastatic spread of pancreatic cancer cells. Our results suggest that the NISCH agonist rilmenidine is a promising candidate for drug repurposing as an antimetastatic agent in PDAC, and that NISCH can be a potential target for the development of new PDAC therapeutics.

## 1. Introduction

Pancreatic cancer is one of the most lethal cancers, with a five-year survival rate of 13% [1]. Pancreatic ductal adenocarcinoma (PDAC) accounts for almost 90% of all pancreatic cancer cases [2]. With clinically silent early lesions, PDAC is generally diagnosed at an already advanced stage when it has metastasized to other organs, and the treatment options are limited [3]. Only 20% of PDAC patients are amenable to potentially curable surgical removal [4]. Unfortunately, even in patients with upfront resection and adjuvant therapy, the five-year survival rate that can be expected is only 19% [5]. Metastatic dissemination in PDAC starts very early [6], and it is estimated that only five years are required for the initial non-metastatic founder cell to acquire the metastatic ability [7]. Despite improvements in therapeutic approaches, PDAC remains poorly responsive to chemotherapy, targeted therapy, and immunotherapies, and currently, there are no specific approaches for targeting the metastatic disease.

Fibrosis is the main feature of PDAC, and the dense stroma—consisting of the extracellular matrix, sparse immune infiltrate, cancer-associated fibroblasts (CAFs), and tumor vasculature—can comprise up to 90% of the tumor mass [8]. CAFs, with their heterogeneous nature, participate in various signaling pathways that contribute to tumor progression [9]. Their role in the modification of the extracellular matrix [10], cancer cell migration and invasion processes [11], and drug resistance [12] has raised awareness about the necessity of examining the effects of systemically delivered drugs not only on cancer cells but also on the tumor microenvironment (TME).

Nischarin (NISCH) is a cytosolic protein, first identified in mice as a binding partner of integrin α5, involved in the regulation of cell motility [13]. Soon after, it was recognized that it was homologous to the IRAS-I1 receptor antisera-selected protein in humans [14]. NISCH was shown to have tumor suppressor properties in breast cancer and to modulate cell migration and invasion through interactions with numerous signaling proteins, such as Rac Family Small GTPase 1 (Rac1), p21-activated kinase 1 (PAK1), LIM domain kinase 1 (LIMK1), and Liver Kinase B1 (LKB1) [15]. Our group recently examined NISCH expression across human cancers and found that it was decreased in tumor tissues compared to their healthy counterparts in most solid tumor types, and (based on the gene set enrichment analysis) that it was negatively associated with pathways that control cancer growth and progression [16].

Of importance, there are several clinically approved drugs that bind to IRAS-I1/NISCH [17], out of which the antihypertensive rilmenidine has been reported as the most potent agonist [18]. NISCH expression and its role in pancreatic cancer have not been investigated in detail. Given the tumor-suppressive properties of NISCH, we hypothesized that its agonist, rilmenidine, might affect PDAC tumor growth and/or invasion and that it could have the potential for drug repurposing in PDAC treatment. Here, we present findings on the anti-metastatic effects of rilmenidine on PDAC cancer cells and their interaction with the microenvironment.

## 2. Materials and Methods

### 2.1. Acquisition and Processing of Data on NISCH Expression from Public Repositories

The Cancer Genome Atlas (TCGA) pancreatic adenocarcinoma dataset was downloaded from the web-based bioinformatics tool cBioPortal (https://www.cbioportal.org/study/summary?id=paad_tcga_pan_can_atlas_2018, accessed on 2 February 2021) and merged with TCGA Pancreatic adenocarcinoma (PAAD) data from the Morpheus website (https://software.broadinstitute.org/morpheus/, accessed on 2 February 2021) to obtain the transcriptomic values of normal samples [19,20]. Pancreatic adenocarcinoma (QCMG, Bailey) dataset was also obtained from the web-based bioinformatics tool cBioPortal (https://www.cbioportal.org/study/summary?id=paad_qcmg_uq_2016, accessed on 7 April 2026) [21]. PDAC datasets GSE16515 [22] and GSE62452 [23] from the NCBI Gene Expression Omnibus (GEO) database were downloaded in pre-processed form [24] and raw RNA-seq results of the GSE93326 dataset [25] were pre-processed in R. GEO datasets were uploaded to the Gene Set Enrichment Analysis (GSEA) Broad Institute software, version 4.3.3 [26] to collapse datasets from probes to symbols using the max_probe collapsing mode.

NISCH expression in normal pancreas and PDAC samples from the GSE16515 and GSE62452 datasets, and matching stromal and epithelial samples from the GSE93326, were analyzed with a paired *t*-test using GraphPad Prism 7. Patients from databases containing information about survival (TCGA PAAD and GSE62452) were divided into the NISCH high and NISCH low expression groups based on the mean NISCH expression of the entire population, and survival analysis was performed using the log-rank (Mantel–Cox) test. The HPA database (https://www.proteinatlas.org/ENSG00000010322-NISCH/pathology/pancreatic+cancer, accessed on 2 July 2024) [27] was used to download images of tumor tissues stained with HPA023189 antibody. GEPIA (http://gepia.cancer-pku.cn/, accessed on 2 July 2024) [28] was used to examine the mRNA expression of NISCH at different stages of PDAC and to generate box plots. The expression level of total NISCH protein in normal pancreatic tissue and pancreatic adenocarcinoma from the Clinical Proteomic Tumor Analysis Consortium (CPTAC) was plotted with a Jitter plot using the UALCAN web tool [29] (accessed on 30 July 2024).

For infiltration estimation for PAAD TCGA tumors, TIMER2.0 website (http://timer.cistrome.org/, accessed on 2 July 2024) was used [30]. We used Gene module to visualize the correlation of NISCH expression with the immune infiltration level of B cells, CD8+, CD4+, dendritic cells, macrophages, and neutrophils.

### 2.2. Immunohistochemistry

The Human Pancreatic Tumor tissue microarray (TMA) was purchased from Novus Biologicals (NBP2-78128, Centennial, CO, USA). The tissue array included 24 formalin-fixed cases of normal, reactive, and cancerous pancreatic tissues. Staining was performed as previously described [31]. Details of the primary antibodies used are shown in the Supplementary Table S1.

For the immunofluorescent staining, secondary antibodies, goat anti-rabbit IgG (H+L) Cyanine3 (dilution 2 μg/mL, A10520, Invitrogen, Waltham, MA, USA) and goat anti-mouse Alexa Fluor 488 secondary antibody (37120, Invitrogen) were used. Nuclei were stained with DAPI (dilution 1:1000, 90229, Merck Millipore, Burlington, MA, USA), and slides were mounted with Antifade mounting medium (AR0036, Boster Biological Technology, Pleasanton, CA, USA). Images were acquired on a Zeiss PALM MicroBeam Axio Observer Z1 microscope (Carl Zeiss MicroImaging GmbH, Jena, Germany) with a Zeiss LD Plan-Neofluar 20×/0.4 Korr, ∞/0–1.5 mm lens and AxioVision SE64 Rel4.9.1 software.

### 2.3. Cell Lines and Culture Conditions

PANC-1 and MIA PaCa-2 cell lines and the Pancreatic Cancer Panel ATCC-TCP-1026 were obtained from the American Type Culture Collection (ATCC, Manassas, VA, USA) at the beginning of this study and were not additionally authenticated. Cells were grown in media recommended by the ATCC. All culture media were supplemented with 10% fetal bovine serum (FBS) (Sigma-Aldrich, Burlington, MA, USA) and an antibiotic solution (PS-B, Capricorn Scientific, Ebsdorfergrund, Germany). All cell lines were occasionally tested for the presence of mycoplasma by PCR [32], and if required, they were treated with MycoXpert (Capricorn Scientific).

Primary human CAFs were propagated from three different patients (labeled as CAF1, CAF2, and CAF3, all derived from female patients aged 63–74 years, therapy naïve). Written informed consent was obtained from all the patients. CAFs were isolated using the outgrowth method described by Bachem et al. [33]. CAF cultures were tested for the absence of E-cadherin and Cytokeratin 19 by immunocytochemistry. CAFs were not immortalized and spontaneously reached senescence around the 25th passage, which limited the number of assays that we were able to perform on each CAF line (Supplementary Table S2 lists the assays performed on CAF cell lines).

### 2.4. Immunoblotting

Cellular proteins were extracted using Pierce RIPA cell lysis buffer (89901, ThermoFisher Scientific, Waltham, MA, USA) with the addition of phosphatase (4906837001, Roche, Basel, Switzerland) and protease (5892791001, Roche, Basel, Switzerland) inhibitors. Protein concentration was measured using a BCA protein assay kit (23225, Thermo Scientific, Waltham, MA, USA); 20–30 μg of lysates were separated on 8% SDS-PAG electrophoresis gels, and proteins were transferred onto PVDF membrane (88518, ThermoFisher Scientific). After the blocking step, membranes were incubated with primary antibodies at 4 °C overnight (details about primary antibodies can be found in Supplementary Table S1). Horseradish peroxidase (HRP)-linked goat anti-mouse IgG secondary antibody (#7076, Cell signaling, Danvers, MA, USA) or HRP-linked goat anti-rabbit IgG secondary antibody (#7074, Cell signaling) were used at a 1:2000 dilution, and membranes were incubated for 1 h at RT. Signal was developed with SuperSignal™ West Pico PLUS Chemiluminescent Substrate kit (34580, ThermoFisher Scientific) and imaged on Azure600 Blot Imaging System (AZI600-01, Azure Biosystems, Dublin, CA, USA). Protein band intensities were quantified using ImageJ software 1.53e by measuring the integrated density values and normalizing to the loading control. Results were expressed relative to the untreated sample.

### 2.5. Drug Treatments and MTT Viability Assay

Rilmenidine hemifumarate (R-134), moxonidine hydrochloride (M1559), and clonidine hydrochloride (C7897) were procured from Sigma-Aldrich and dissolved in DMSO (dimethyl sulfoxide) according to the manufacturer’s instructions.

MTT assay was performed after 72 h of treatment in the concentration range from 0 to 600 μM, as described previously [16]. DMSO was used in controls, where 0.2% DMSO corresponded to the concentration used in the 100 μM rilmenidine treatment, 0.7% for 300 µM rilmenidine, and 1.4% for 600 µM rilmenidine.

### 2.6. Apoptosis Assay

PANC-1 and MIA PaCa-2 cells were seeded at 1.5 × 10^5^ cells/well, and BxPC-3 at 3 × 10^5^ cells/well in 6-well plates. Twenty-four hours after the seeding, the cells were treated and incubated with the rilmenidine for an additional 24, 48, or 72 h. Cell death was analyzed using an Annexin V-fluorescein isothiocyanate apoptosis detection kit, according to the manufacturer’s instructions (556419, BD Biosciences, San Jose, CA, USA) on a FACS-Calibur cytometer with Cell Quest computer software (version 6.0, Becton Dickinson, Franklin Lakes, NJ, USA).

### 2.7. RNA Sequencing and Gene Ontology (GO) Enrichment Analysis

Total RNA from three independent experiments of control and 100 μM rilmenidine-treated MIA PaCa-2 cells at the time point of 24 h, and from two independent experiments for the time point of 48 h, was isolated using TRIzol reagent (15596018, Life Technologies, Carlsbad, CA, USA), and concentration was measured on BioSpec-nano Spectrophotometer (Shimadzu Biotech, Kyoto, Japan). Samples were prepared at a final concentration of 20 ng/μL in water and sequenced at Novogene Co. (Cambridge, UK), which provided Gene Ontology (GO) enrichment analysis of differentially expressed genes and a graphic representation of results. It was implemented by the cluster Profiler R package, in which gene length bias was corrected, and GO terms with a corrected *p*-value of less than 0.05 were considered to be significantly enriched. Differential expression analysis of two conditions/groups (two or three biological replicates per condition) was performed using the DESeq2 R package (1.20.0). The resulting *p*-values were adjusted using Benjamini and Hochberg’s approach for controlling the false discovery rate. Genes with the adjusted *p*-value ≤ 0.05 found by DESeq2 were assigned as differentially expressed. The genes were ranked according to the degree of differential expression in the two samples, and then the predefined Gene Sets were tested to see if they were enriched at the top or bottom of the list. Raw sequencing data of MiaPaca-2 cell line mRNA transcriptome, treated for 24 or 48 h in vitro with 100 μM rilmenidine, can be found at https://zenodo.org/records/6920520 (accessed on 2 July 2024) and https://zenodo.org/records/6948536 (accessed on 2 July 2024), respectively.

### 2.8. Cell Spreading Assay

PANC-1, MIA PaCa-2, and BxPC-3 cells were seeded at 5 × 10^3^ cells/well in 48-well plates (Corning, Corning, NY, USA), uncoated or pre-coated with 2 μg/cm^2^ fibronectin (F4759, Sigma-Aldrich) or collagen type I (C3867, Sigma-Aldrich). Cells were treated with rilmenidine and imaged at 4, 6, 8, and 24 h post-seeding. Cells were seeded in triplicate wells, and images were taken in 2 fields of view per well, with a total of 6 fields analyzed per condition. Phase-bright round cells were counted as non-spread; cells with any protrusions were counted as spread. Experiments were performed three times.

### 2.9. Nischarin Knockdown

All transfections were performed in 6-well culture dishes using 1 μg DNA with Optimem and Lipofectamine 2000 reagents (Invitrogen) as per the manufacturer’s instructions. MIA PaCa-2 cells were individually transfected with 5 unique 29mer shRNA constructs in pRFP-C-RS vector (four sh NISCH and one sh scramble) from the OriGene NISCH Human shRNA Plasmid Kit (#TF311169, Origene, Rockville, MD, USA). The cells were then grown for 72 h to allow cell recovery and puromycin resistance expression before selection with 1.25 μg/mL puromycin (IC90 determined by MTT of the parental line). The level of knockdown was determined by Western blot. Clone transfected with construct 2 (named shNISCH-2 in the manuscript) was used in wound healing assays.

### 2.10. Wound-Healing Assay

PANC-1, MIA PaCa-2, and BxPC-3, or MIA PaCa-2 scramble (scr) and NISCH knockdown clone sh2 (shNISCH-2) cells were seeded in 6-well plates at 5 × 10^5^ cells/well in growth media supplemented with 10% FCS. 24 h after seeding, the wound was made with a 200 μL pipette tip, and detached cells were washed with PBS solution. To decrease the proliferation of cells and ensure that the closing of the wound was a consequence of the directional migration of cells, treatment was performed in a medium supplemented with 1% FCS. Images of at least two areas per well were taken at 0 and 24 h after rilmenidine treatment at 10X magnification (Olympus CX33, Hamburg, Germany). The closure area of the migrating cells was calculated using ImageJ software (1.53e, National Institutes of Health, Bethesda, MD, USA), and the percentage of wound closure was calculated at 24 h post-wounding, normalized to untreated control.

### 2.11. Transwell Assays

For transwell migration, 5 × 10^4^ cells/insert were seeded in 0.8 μm pore inserts (353097, Corning Scientific, Corning, NY, USA) in the presence of rilmenidine in serum-free medium. The lower chamber medium was supplemented with 10% FCS as a chemoattractant. Cells were incubated for 48 h. For the transwell invasion assay, inserts were pre-coated with 2 µg/cm2 fibronectin (F4759, Sigma-Aldrich), 10 µg/mL collagen type I (C3867, Sigma-Aldrich) or 0.4–0.6 mg/mL Cultrex^®^ Reduced Growth Factor Basement Membrane Extract (RGF-BME, 3433-005-01, R&D Systems, Minneapolis, MN, USA) and left to invade for 72 h. Cells that migrated or invaded the pores were fixed with 3.7% formaldehyde and stained with 0.5% Crystal Violet Dye in 25% methanol. Images of migrated and invaded cells were taken at 10× magnification (Olympus CX33) in at least three independent fields of view.

### 2.12. Active Rac1 Pull-Down Assay

Rac1 activity was measured using RhoA/Rac1/Cdc42 Activation Assay Combo Biochem Kit (#BK030, Cytoskeleton, Denver, CO, USA) following the manufacturer’s protocol. Briefly, PANC-1 cells were seeded in 10 cm Petri dishes at a density of 1.5 × 10^6^ and left overnight at 37 °C and 5% CO_2_ to attach. Multiple wounds were made in the cell monolayer using a 200 μL pipette tip, and after the PBS wash medium was changed to DMEM with 1% FCS with or without rilmenidine. The 24 h post-wounding cells were collected using the Cell lysis buffer supplemented with protease inhibitor cocktail. Protein concentration was determined using the BCA protein assay kit, and 600 μg of total cell protein was added to PAK-PBD beads. After incubation and wash steps, 20 μL of 2× Laemli Sample Buffer (#1610747, Bio-Rad, Berkeley, CA, USA) was added to each tube, and samples were boiled for 2 min at 95 °C. Samples were analyzed by immunoblotting with anti- Rac1 monoclonal antibody as per the manufacturer’s recommendations.

### 2.13. Gelatin Zymography

PANC-1, MIA PaCa-2, and BxPC-3 cells (2 × 10^6^/tissue culture dish) were seeded in the presence of rilmenidine in serum-free media and were incubated for another 6, 24, and 48 h. After the incubation, conditioned media from the cell culture were collected and further concentrated by using the protein concentrator spin columns (88517, ThermoFisher Scientific). Cells were lysed by scraping on ice with 200 µL of Cell Lysis Buffer (K490-100-2, BioVision, Milpitas, CA, USA), centrifuged, and the supernatant was collected for further analysis. Protein quantification was performed by the BCA method. Gelatin zymography of conditioned media samples and cell lysates was performed on 8% SDS-PAGE containing 1 mg/mL of gelatin (48723, Sigma-Aldrich) under non-reducing conditions, as previously described [34]. After rinsing in renaturing buffer (2.5% Triton X-100), the gels were soaked in the developing buffer containing 50 mM Tris-HCl, 200 mM NaCl, 5 mM CaCl_2_, and 0.05% NaN_3_, pH 7.5, at 37 °C for the next 48 h. Gels were then stained with 0.5% Coomassie brilliant blue for 1 h and destained in 40% methanol and 10% acetic acid. An additional destaining step with 1% Triton X-100 was performed to obtain sharper MMP bands over the blue background. The transparent bands corresponding to MMP activity were analyzed by optical densitometry with ImageJ software.

### 2.14. Immunofluorescence Staining

CAFs were seeded in 96-well optical plates (165305, ThermoFisher Scientific) at a density of 2 × 10^3^ cells/well. Cells were treated with 100 μM rilmenidine 24 h after seeding and incubated for another 72 h, after which they were fixed with 3.7% formaldehyde for 15 min, permeabilized with 0.2% Triton X-100 (T-6878, Sigma-Aldrich) on ice for 2 min, and blocked with 1.5% BSA for 30 min at RT. Fixed cells were incubated in primary antibodies overnight at 4 °C (Supplementary Table S1). Secondary antibodies were added at a 1:250 dilution: goat anti-mouse Alexa Fluor 488 (37120, Invitrogen), and goat anti-rabbit Alexa Fluor 488 secondary antibody (dilution 4 μg/mL, A11008, Invitrogen) and incubated for 1 h, at RT. Actin fibers were stained with Phalloidin TRITC Dye (dilution 1:500, 90228, Merck Millipore) for an hour, and nuclei were stained with DAPI for 2 min. Cells were imaged on a fluorescent microscope Zeiss PALM MicroBeam Axio Observer Z1 using 10×, 20× or 63× Zeiss LD Plan-Neofluar objectives.

### 2.15. Co-Culture of Pancreatic Cancer Cells and CAFs

CAFs were seeded in 6-well plates at a density of 5 × 10^5^ cells/well in DMEM supplemented with 10% FCS. Inserts with 0.4 μm pore size (CC Insert MD6 140640, ThermoFisher Scientific) were placed in the wells, and PANC-1 cells were seeded into the inserts at a density of 3 × 10^5^ cells/insert. After 24 h, the medium was replaced in both compartments with DMEM without serum, with or without rilmenidine, and the co-cultures were incubated for another 72 h. Conditioned medium was collected for dot blot and ELISA analyses, and cell pellets were collected for RT-PCR analysis.

### 2.16. Dot-Blot Analysis

Conditioned media from the rilmenidine-treated and untreated co-cultures of PANC-1 cells with CAF1 or CAF2 cells were collected. The human cytokine array (ARY005B, R&D Systems) was used to determine differences in cytokine expression between treated and untreated samples. The experiment was performed according to the manufacturer’s instructions. Briefly, conditioned media were first concentrated on Amicon^®^ Ultra-4 Centrifugal Filter Units (UFC800308, Merck Millipore) for 20 min, and total protein concentration was determined using the BCA assay. The volumes of samples used in the reaction were 400 μL for media from PANC-1—CAF1 co-culture condition and 200 μL for media from PANC-1—CAF2 co-culture condition. Human Array Detection Antibody Cocktail (15 μL) was added to dot-blot membranes and incubated overnight at 4 °C. After washing, membranes were incubated in Streptavidin-HRP conjugate for 30 min at room temperature. For the detection, Chemi Reagent Mix was used, and images were acquired on the Azure600 Blot Imaging System. The integrated intensity of the dots was determined using Image J 1.53e software and normalized to the amount of the loaded protein. Cytokines for which the difference in amount was observed were plotted in GraphPad Prism 8.0.1 as integrated density per μg of protein.

### 2.17. Enzyme-Linked Immunosorbent Assay

Conditioned media from co-cultures of PANC-1 or MIA PaCa-2 cells and CAF2 cells were collected as described above. The concentration of total proteins was determined in the BCA assay. Interleukin-6, interleukin-8, and PAI-1 levels were detected using a commercial Human IL-6 ELISA kit (88-7066-22, Invitrogen), Human IL-8 ELISA kit (88-8086, Invitrogen) and Human SERPINE1 (Plasminogen activator inhibitor 1) ELISA kit (EH0538, FineTest, Wuhan, China), respectively. ELISAs were performed according to the manufacturer’s instructions. Absorbance was measured at 450 nm using a microplate reader. After measuring the absorbance, concentrations were determined based on the standard curve, and the results were normalized to the concentration of total loaded proteins.

### 2.18. Analysis of Gene Expression by Real-Time PCR

Total RNA was isolated using TRIzol reagent and was reverse transcribed into complementary DNA using a High Capacity cDNA RT kit (4368814, ThermoFisher Scientific) as per the manufacturer’s instructions. The qPCR was performed using SYBR Green Master Mix (A25742, Applied Biosystems, Foster City, CA, USA) and gene expression was calculated by the 2^−ΔΔ*Ct*^ method. Sequences of the primers used for analysis are shown in Supplementary Table S3.

### 2.19. Zebrafish Pancreatic Adenocarcinoma Xenograft Model

All procedures involving zebrafish (*Danio rerio*) embryos complied with the European Directive 2010/63/EU and adhered to the Ethical Guidelines for the Care and Use of Laboratory Animals established by the Institute of Molecular Genetics and Genetic Engineering, University of Belgrade. Wild-type zebrafish embryos (AB strain), kindly provided by Dr. Ana Cvejić (Wellcome Trust Sanger Institute, Cambridge, UK), were reared, and the toxicity of rilmenidine was evaluated in accordance with the general principles of the OECD guidelines for chemical testing, as previously described [35]. Rilmenidine stock was diluted directly in E3 medium (consisting of 5 mM NaCl, 0.17 mM KCl, 0.33 mM CaCl_2_ and 0.33 mM MgSO_2_ in distilled water) for exposure. DMSO (0.25%) and E3 medium alone were used as the negative controls. The treated embryos were examined daily under a stereomicroscope (Carl Zeiss™ Stemi 508 doc, Germany) for the appearance of apical endpoints up to 120 hpf (Supplementary Table S4). The experiment was performed in triplicate, with 20 embryos per concentration. At 120 hpf, embryos were anesthetized by the addition of 0.1% (*w*/*v*) Tricaine solution (Sigma-Aldrich), photographed, and euthanized by freezing at −20 °C for ≥24 h.

For the xenograft experiments, PANC-1 cells were resuspended in serum-free DMEM and labeled with 2 μM Red CellTracker fluorescent dye according to the manufacturer’s instructions. Approximately 5 nL of the suspension containing 150 labeled PANC-1 cells was microinjected into the yolk sac of transgenic Tg(*fli1*:EGFP) zebrafish embryos at 48 hpf using a pneumatic picopump (PV820, World Precision Instruments, Sarasota, FL, USA). The injected embryos were incubated at 28 °C for one hour to recover and examined under a fluorescence microscope to confirm the presence of fluorescently labeled tumor cells. Four hours post microinjection, successfully xenografted embryos were treated with rilmenidine and maintained at 33 °C until the 120 hpf stage (3 days of treatment). The control group comprised xenografted embryos treated with 0.25% DMSO. The treated embryos were examined daily by fluorescence microscopy to evaluate the effect of rilmenidine on the survival of PANC-1 xenografts, the number of xenografts with metastases, and the metastatic cell dissemination rate. The experiment was repeated twice with 10 embryos per treatment.

### 2.20. Statistical Analysis

The number of experimental repeats for each assay is shown in the figure legends, and all results were expressed as the mean of technical triplicates or mean ± SD. Statistical analysis was performed using GraphPad Prism 8.0.1 (GraphPad Software Inc., San Diego, CA, USA). Differences between control and treatment groups were analyzed with paired or unpaired two-tailed Student’s *t*-test and one-way or two-way ANOVA, with a *p* < 0.05 considered as statistically significant.

## 3. Results

### 3.1. Nischarin Is Expressed in PDAC and Is a Favorable Prognostic Marker

**Figure 1:**
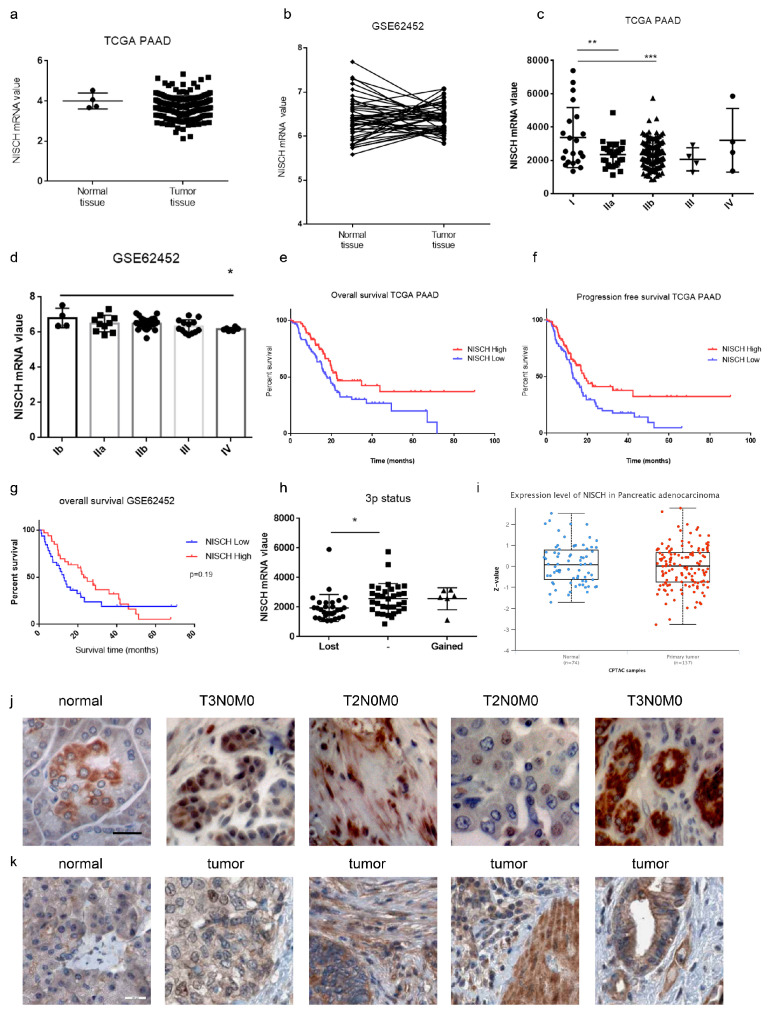
Nischarin is expressed in PDAC. (**a**) *NISCH* mRNA expression in normal and tumor pancreatic tissues from the TCGA PAAD cohort; (**b**) *NISCH* mRNA expression in the paired tumor and adjacent tissue samples in the GSE62452 cohort; (**c**) *NISCH* mRNA expression by stage in the TCGA PAAD cohort, ** *p* = 0.0052, *** *p* = 0.0003, adjusted *p*-value, one-way ANOVA, Tukey’s multiple comparison test, and (**d**) the GSE62452 cohort, * *p* = 0.0414, one-way ANOVA, Tukey’s multiple comparison test; (**e**) Kaplan-Meier plots for the overall and (**f**) progression free survival of PDAC patients in the TCGA PAAD cohort divided into two groups using the best cut-off value for NISCH. *p* = 0.01 and *p* = 0.018 by Mantel-Cox test, respectively; (**g**) The overall survival of patients in the GSE62452 cohort (*p* = 0.19 by Mantel-Cox test); (**h**) *NISCH* mRNA expression in groups based on the status of the 3p chromosome, in patients from the TCGA PAAD cohort, * *p* = 0.0299, one-way ANOVA, Tukey’s multiple comparison test; (**i**) Total NISCH protein level in normal and cancer pancreatic tissue from CPTAC; (**j**) NISCH expression in the normal and tumor pancreatic tissue from the NBP2-78128 microarray, scale bar 20 μm; (**k**) NISCH expression in the normal and tumor pancreatic tissue deposited in the Human Protein Atlas (HPA023198), scale bar 20 μm.

First, we examined NISCH mRNA and protein expression in publicly available databases. NISCH mRNA and protein were expressed in both healthy and tumor pancreatic tissues in the HPA, and there was no difference in the mRNA levels between healthy and cancer tissues in the TCGA PAAD dataset (Figure 1a) nor in the paired tumor and adjacent tissue samples in the GSE62452 (Figure 1b) and GSE16515 datasets (Supplementary Figure S1a). In the TCGA PAAD cohort, stage IIa and IIb tumors had lower *NISCH* mRNA expression than stage I tumors (Figure 1c), while in the GSE62452 cohort, only stage IV tumors had lower expression than stage I tumors, as determined by Tukey’s multiple comparisons test (Figure 1d). There was no difference in expression based on the tumor grade or between male and female patients (Supplementary Figure S1b,c). Using the mean expression cut-off for separation into high and low *NISCH* expression groups, we found that *NISCH* was a positive prognostic marker in terms of both progression-free and overall survival of PDAC patients in the TCGA PAAD cohort (*p* = 0.01 and *p* = 0.018, by Mantel-Cox test, respectively) (Figure 1e,f). In the GSE62452 cohort, patients with higher *NISCH* mRNA expression had longer survival, although the difference did not reach significance (*p* = 0.19, Mantel-Cox test, Figure 1g). Additionally, in the Bailey data set [21] patients with higher *NISCH* mRNA expression had longer overall survival (*p* = 0.0015, Mantel-Cox test, Supplementary Figure S1d). Taken together, higher *NISCH* expression in PDAC was associated with longer overall survival.

The *NISCH* gene is located on the 3p21.1 chromosome, a location marked as a putative tumor suppressor cluster [36]. In the TCGA PAAD dataset based on the 3p status, tumors with 3p loss had significantly lower *NISCH* expression (Figure 1h).

In the CPTAC database, mass-spectrometry-based proteomic data on NISCH protein expression were available on healthy and tumor tissue; however, NISCH was not significantly lower in the tumor compared to the normal pancreas (Figure 1i). Next, we examined NISCH protein localization by immunohistochemistry (anti-nischarin #558262, BD Pharmingen) in the PDAC tissue microarray (NBP2-78128, Novus Biologicus Cambridge, UK). Nischarin was expressed in normal pancreatic tissue and in most of the examined pancreatic cancer tissue samples (Figure 1j). Intriguingly, in addition to the membranous and cytoplasmic localization, we observed NISCH in the nuclei of cells in the tumor tissues. To further corroborate this, we examined NISCH expression and assigned localization in the HPA, in which samples were stained with a different antibody (HPA023198). Indeed, in the HPA, NISCH expression in PDAC was described as membranous, cytoplasmic, and nuclear (in 50% of the samples) (Figure 1k). Importantly, in the tissue microarray we examined, as well as in the HPA, NISCH was observed in both the epithelial and stromal compartments of the tumor.

### 3.2. NISCH Is Expressed in Both the Epithelial and Stromal Compartments of PDAC

**Figure 2:**
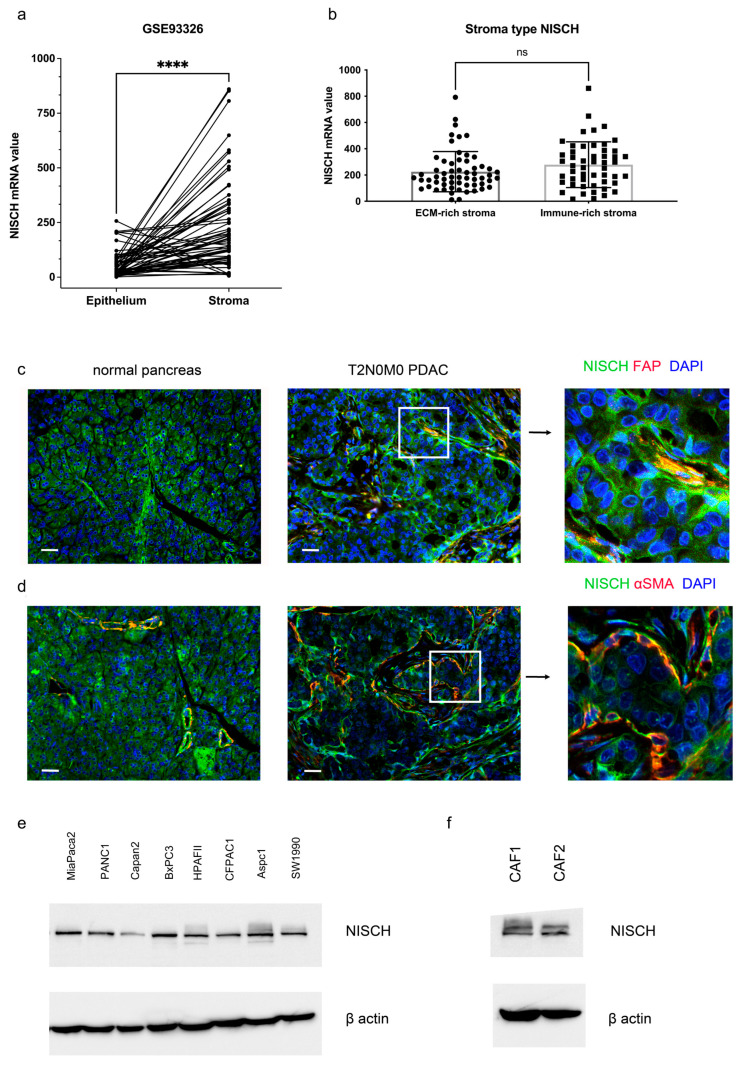
Nischarin is expressed in both the epithelial and stromal compartments of PDAC. (**a**) *NISCH* mRNA expression in paired epithelial and stromal compartments of PDAC from the GSE93326 cohort, **** *p* < 0.0001, two-tailed paired *t*-test; (**b**) *NISCH* mRNA expression in the ECM-rich and immune-rich type stroma in the GSE93326 cohort, ns = non-significant (*p* ≥ 0.05); (**c**) NISCH (green) and FAP (red) localization in the normal and tumor pancreatic tissue from the NBP2-78128 microarray, nuclei blue; scale bar 100 μm (**d**) NISCH (green) and αSMA (red) localization in the normal and tumor pancreatic tissue from the NBP2-78128 microarray, nuclei blue; scale bar 100 μm (**e**) NISCH protein expression in PDAC cancer cell lines, and in (**f**) patient-derived CAFs.

Any systemic therapy for PDAC could potentially reach all the components of the tumor tissue, both the cancer and stromal compartments. To infer *NISCH* mRNA localization, we examined data from the GSE93326 dataset [25], which has paired laser-microdissected epithelial and stromal compartment samples from 65 patients. Intriguingly, *NISCH* mRNA expression was higher in the stromal than in the epithelial compartment (*p* = 0.0001 by paired *t*-test) (Figure 2a), but there was no difference in expression between the ECM-rich and immune-rich stromal compartments (Figure 2b). To validate this finding, we co-stained for NISCH with two CAF markers, fibroblast activation protein (FAP) and alpha smooth muscle actin (αSMA) [8], in the TMA. We found that NISCH was expressed in both FAP (Figure 2c) and αSMA-positive cells (Figure 2d). In line with our findings, based on the Timer estimate, *NISCH* expression was not associated with tumor purity in PDAC and partially correlated with various immune cell infiltrates (Supplementary Figure S1e). We stained for NISCH and CD45 in the TMA but found very few CD45-positive infiltrates in the samples, except for the liver metastasis sample (Supplementary Figure S1f). Finally, we examined NISCH expression in a panel of human PDAC cell lines representing both classical and quasimesenchymal phenotypes (MIA PaCa-2, PANC-1, Capan-2, BxPC-3, HPAF-II, CFPAC-1, AsPC-1, and SW 1990) and in CAFs isolated from PDAC tumor tissue. NISCH was expressed in all the tested cancer cell lines (Figure 2e) and CAFs (Figure 2f). These results confirm that NISCH is expressed in both epithelial and stromal compartments of PDAC tissue, and imply that any drug targeting NISCH would have an impact on both cancer cells and stroma.

### 3.3. Nischarin Agonists Affect Pancreatic Cancer Cell Fitness and Induce Significant Transcriptional Changes In Vitro

**Figure 3:**
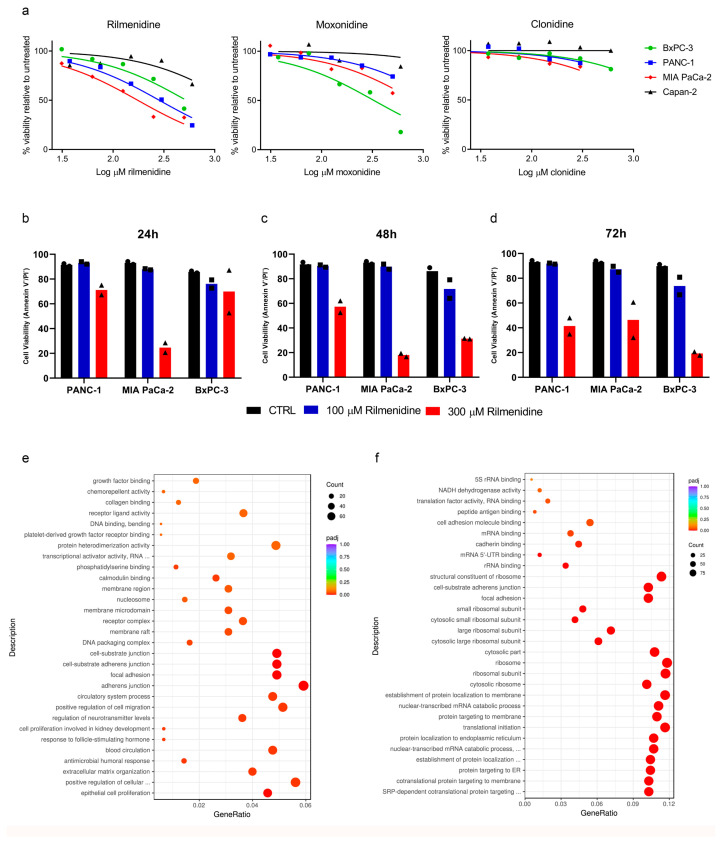
Effects of NISCH agonist treatment on PDAC cells in vitro. (**a**) Percent viability of PDAC cells after 72 h of treatment with increasing concentrations of rilmenidine, moxonidine or clonidine determined by MTT assay, presented as log(inhibitor) vs. normalized response. Experiment was performed in triplicates, with at least three biological repeats. (**b**) The proportion of live Annexin V-/PI- cells in the population of untreated and cells treated with 100 and 300 μM rilmenidine for 24 h, (**c**) 48 h, or (**d**) 72 h. Results are shown as % of viable (Annexin V-/PI-) cells; (**e**) Gene Ontology functional gene expression enrichment analysis of MIA PaCa-2 cells treated with 100 μM rilmenidine for 24 h or (**f**) 48 h.

To examine the effects of I_1_-Imidazoline receptor IRAS-I1/NISCH agonists on cancer cell fitness, we selected the cell lines derived from the primary tumor site—MIA PaCa-2, PANC-1, Capan-2, and BxPC-3—and tested the effects of rilmenidine and moxonidine (IRAS-I1 selective agonists from the second generation of central antihypertensive drugs) and clonidine (the first-generation non-selective IRAS-I1 agonist). We performed the MTT viability assay after 72 h of treatment with increasing drug concentrations (0–600 μM). We found that rilmenidine, with the highest affinity for NISCH of the three tested drugs [17], had the most potent effect in all four tested cell lines, while clonidine had no effect on cell viability (Figure 3a). The least sensitive cell line to all three drugs was Capan-2, which expressed the lowest amount of NISCH (Figure 2e). We selected rilmenidine as the most potent compound for further investigation and examined the sensitivity of the rest of the PDAC cell lines from the panel (IC_50_ values, Supplementary Table S5). The MIA PaCa-2 cell line was the most sensitive to the treatment, with an IC_50_ value of 169.2 μM. As the MTT test is based on the reduction of the formazan dye [37] and the decreased viability observed in this test can be a consequence of the decreased proliferation, increased cell death, or the altered redox state in the cells, we tested the effects of 100 μM and 300 μM rilmenidine treatment on apoptosis in the three primary tumor cell lines sensitive to rilmenidine: MIA PaCa-2, PANC-1, and BxPC-3. Flow cytometry analysis of the percentage of live cells negative for Annexin V/PI staining showed a reduction in cell viability as early as 24 h of treatment with 300 μM rilmenidine in all three cell lines, with no apparent changes under 100 μM rilmenidine treatment at any time point (Figure 3b–d). For the MIA PaCa-2 and PANC-1 cell lines, these results are in line with those of the MTT assay, while in the case of the BxPC-3 cells, cell apoptosis and necrosis occurred at much lower doses of rilmenidine than those predicted by the MTT assay. Taken together, rilmenidine treatment induced PDAC cell death only when administered at high concentrations. To infer other possible effects of NISCH agonization in PDAC cells, we sequenced the transcriptome of the MIA PaCa-2 cell line treated with 100 μM rilmenidine for 24 or 48 h. Based on the Gene Ontology (GO) functional gene set enrichment analysis, 24 h treatment with rilmenidine had the most prominent negative effect on the transcripts associated with focal adhesion, adherens junction, cell-substrate adherence junction, positive regulation of cell migration, and extracellular matrix organization (Figure 3e). These findings are in line with the previously reported tumor-suppressive functions of the NISCH protein [15]. After 48 h of treatment, in addition to cell-substrate interactions and focal adhesion-associated transcripts, pathways associated with translation, ribosome assembly, and protein targeting to various localizations in the cells were negatively associated with treatment (Figure 3f). All of these factors are related to proper cytoskeletal organization and imply that rilmenidine treatment affects cytoskeletal dynamics.

### 3.4. Nischarin Agonist Rilmenidine Inhibits Migration and Metastatic Spread of PDAC Cancer Cells

**Figure 4:**
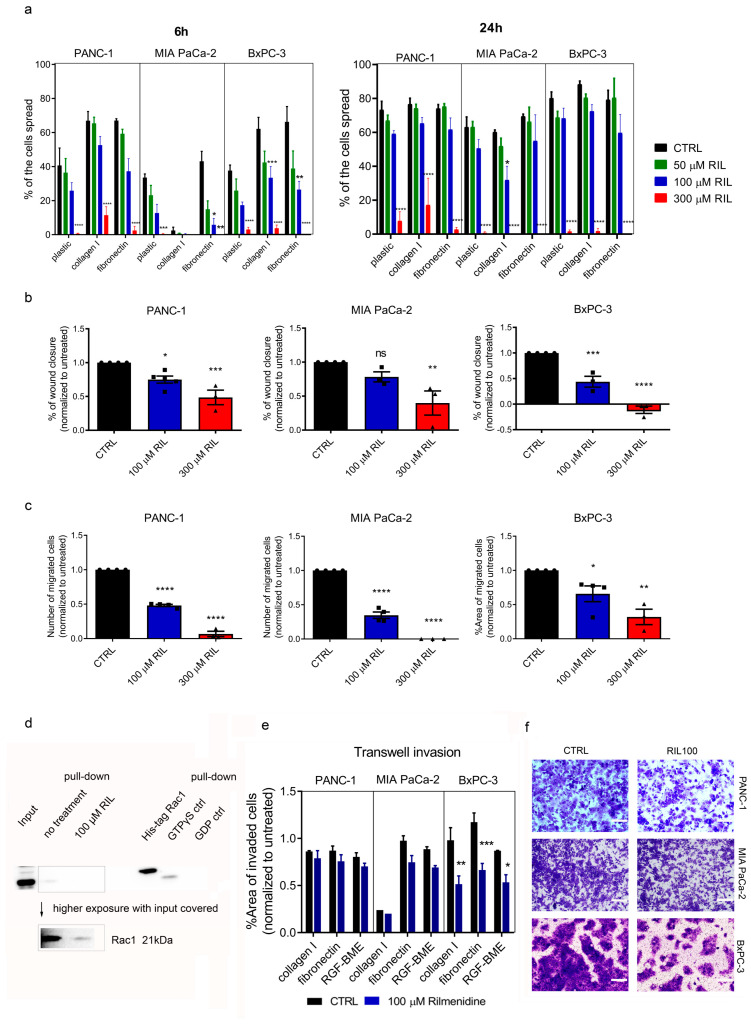
NISCH agonist rilmenidine reduces cell migration and invasion in vitro. (**a**) Quantification of the number of PDAC cells spread 6 and 24 h after seeding on tissue culture plastic, collagen I or fibronectin-coated surface in the presence of rilmenidine. Results were expressed as the percentage of spread cells out of the total counted per field. (**b**) Quantification of the PDAC cell migration in the wound healing assay after 24 h of treatment with rilmenidine; (**c**) Quantification of the PDAC cell migration in the transwell assay after 48 h of treatment with rilmenidine; (**d**) Level of active GTP-bound Rac1 in PANC-1 cells from the wound healing assay 24 h post wounding in control or 100 μM rilmenidine-treated PANC1 cells, pulled–down from 600 μg lysate treated with 10 μL PAK-PBD beads; 20 ng of His-tagged Rac1 protein control, positive cellular protein control GTPγS loaded lysate, negative cellular protein control GTP loaded lysate; (**e**) Quantification of PDAC cell invasion through collagen I, fibronectin or matrigel in the transwell assay after 72 h of treatment with rilmenidine; (**f**) Representative images of transwell invasion through matrigel coat (right). Scale bar 200 μm. All data are shown as mean ± SD. n = 3; * *p* < 0.05, ** *p* < 0.01, *** *p* < 0.001, **** *p* < 0.0001, two-way ANOVA (Sidak’s multiple comparisons test).

NISCH binds to integrin α5 and is involved in the regulation of focal adhesion assembly during cancer cell migration [13]. Therefore, we first investigated the effect of rilmenidine on cell attachment to plastic, fibronectin-, and collagen-coated surfaces. We found that rilmenidine delayed cell attachment in all examined cell lines regardless of the surface coating, while 300 μM treatment completely prevented cell spreading (shown at 6 h and 24 h in Figure 4a, at all-time points in Supplementary Figure S2a–c; photomicrographs are shown in Supplementary Figure S2d–f, in left panels). Under these conditions where cells were plated with treatments, the highest 300 μM dose of rilmenidine induced apoptosis at 48 h, presumably by anoikis (Supplementary Figure S3). MIA PaCa-2 cell line showed a delay in cell spreading on collagen I compared to other surfaces even in the control conditions (Supplementary Figure S2b), given that it did not express integrin α2 important for attachment on collagen-coated matrices [38]. Next, we examined the effects of rilmenidine treatment on collective cell migration using wound-healing (WH) assay and chemotaxis using transwell (TW) migration assay. Rilmenidine treatment impaired cell migration in both WH and TW assays in all three cell lines (Figure 4b,c), with more potent effects in the TW assay in PANC-1 and MIA PaCa-2 cells, where cells individually migrated. Representative images of the WH assay are shown in Supplementary Figure S2d,e,f, in the right panel. To confirm that rilmenidine effects were NISCH-dependent, we performed shRNA knockdown (KD) of NISCH expression in MIA PaCa-2 cells and were able to select clones with reduced NISCH expression (Supplementary Figure S7a). Although modest, decreased NISCH expression significantly increased the migration of MIA PaCa-2 cells in wound healing assay (Supplementary Figure S7b). In addition, NISCH-KD MIA PaCa-2 cells were less sensitive to the inhibitory effect of rilmenidine in the WH assay (Supplementary Figure S7c).

Activation of Rac1 is crucial for cell spreading [39,40] and one of the first reports on NISCH role in cancer cells was that it strongly inhibits Rac1-driven cell motility and invasion [41]. Therefore, we examined the status of Rac1 activation in rilmenidine treated cells during wound healing. We pulled-down GTP-bound Rac1 in PANC-1 cells with PAK-PBD affinity beads (Rac1 Activation Assay, Cytoskeleton) 24 h after introduction of multiple wounds in a confluent layer of cells in absence or presence of 100 μM rilmenidine. Rilmenidine diminished the pool of GTP-bound Rac1 in PANC-1 cells (Figure 4d) implying that impairment of cell attachment and migration under treatment is mediated by inhibition of Rac1 activity.

Next, to investigate the effects of rilmenidine treatment on cell invasion in vitro, we performed TW invasion assays by coating with three types of matrices: collagen I, fibronectin, or Matrigel (RGF-BME). Rilmenidine (100 μM) significantly reduced cell invasion only in the BxPC-3 cell line, regardless of the coated matrix (Figure 4e quantification, representative images of TW invasion through the Matrigel coat Figure 4f). This result is not surprising, as PDAC cells in vitro are phenotypically plastic in terms of migration mode and can adopt both mesenchymal (adhesion- and MMP-dependent) and amoeboidal (adhesion- and MMP-independent) migration modes [42]. In the mesenchymal mode of invasion, cells degrade the ECM, partly through the secretion and/or activation of matrix metalloproteinases (MMPs) [42]. We examined whether rilmenidine affected the activity of collagenases in the conditioned media using the gelatin zymography assay. Based on the predicted molecular size, we observed that BxPC-3 cells mostly secreted MMP-9, whereas PANC-1 cells secreted mostly MMP-2 (Supplementary Figure S4a,b). The MIA PaCa-2 cell line did not produce MMPs under these culture conditions. Rilmenidine slightly reduced MMP gelatinolysis; however, the changes were not statistically significant (Supplementary Figure S4c). While gelatinolytic activity in BxPC-3 cells increased over time (Supplementary Figure S4d), no significant changes were observed after rilmenidine treatment. Western blotting of the conditioned medium from the BxPC-3 cells showed only a small decrease in the MMP-9 expression after rilmenidine treatment (Supplementary Figure S4e). Taken together, these results imply that the effect of rilmenidine on cell migration and invasion is mostly a consequence of a decrease in cell adhesion properties.

**Figure 5:**
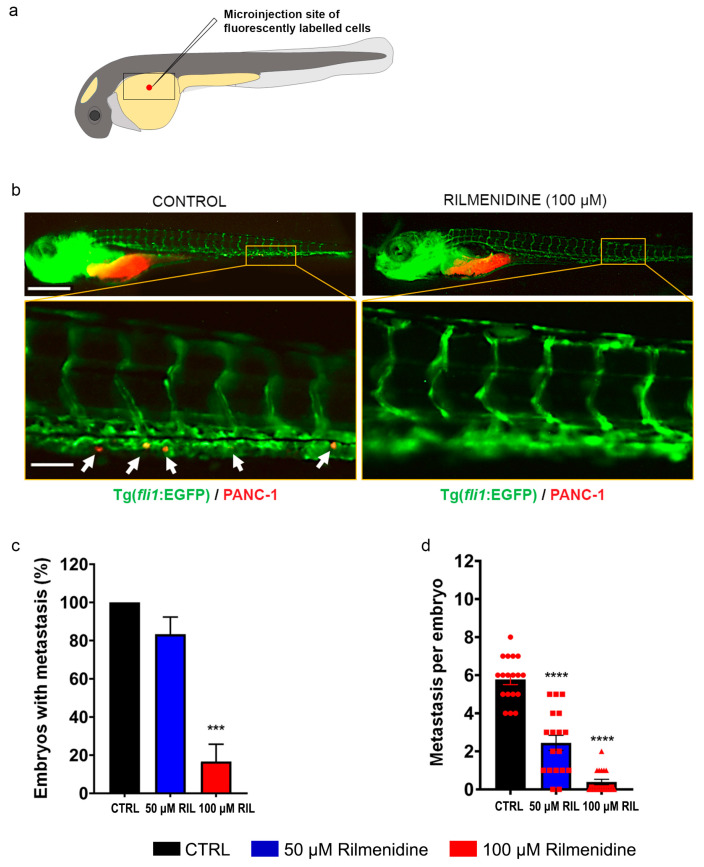
Rilmenidine reduces cell invasion in the Tg(*fli1*:EGFP) zebrafish model. (**a**) Schematic illustration of the microinjection site of fluorescently labelled cells. (**b**) Effects of rilmenidine on PANC-1 cell growth and invasion in Tg(*fli1*:EGFP) zebrafish model. Squares amplify the indicated regions of metastatic sites. Arrowheads point to disseminated PANC-1 cells at 120 hpf. Scale bars 500 µm upper panel, 70 µm lower panel. (**c**) Quantification of the percent of embryos with metastasis and (**d**) total numbers of metastatic foci in the caudal region of zebrafish. The graphs show mean ± SD, *** *p* < 0.001, **** *p* < 0.0001, One-way ANOVA (Dunnett’s multiple comparisons test).

Finally, we used a zebrafish xenograft model to assess the effects of rilmenidine on metastatic dissemination of tumor cells in vivo. This model leverages the optical transparency of zebrafish embryos, enabling real-time visualization of the metastatic spread of PANC-1 cells labeled with CellTracker Red through the bodies of transgenic *Tg*(*fli1*:EGFP) embryos with EGFP-expressing vasculature (Figure 5a,b). As expected for a clinically approved drug, rilmenidine-treated healthy zebrafish embryos showed no visible signs of toxicity, including effects on survival, cardiac function, liver toxicity, and skeletal development (teratogenicity) (list of scored toxicological parameters in Supplementary Table S4). However, in the tumor model, over the course of a 72-hour treatment, rilmenidine significantly inhibited the metastatic dissemination of PANC1 cells throughout the vasculature and body of the treated compared to untreated xenografts. Quantitative analysis revealed that rilmenidine significantly reduced both the proportion of embryos with metastases (100 µM, Figure 5c) and the number of metastatic foci per embryo (50 µM and 100 µM, Figure 5d). These findings demonstrate that treatment with rilmenidine effectively reduced pancreatic cancer cell attachment and migration, leading to a substantial reduction in metastatic spread in the in vivo xenograft model.

### 3.5. Rilmenidine Has an Effect on CAF Phenotype In Vitro and Modulates Cancer Cell-CAF Communication

**Figure 6:**
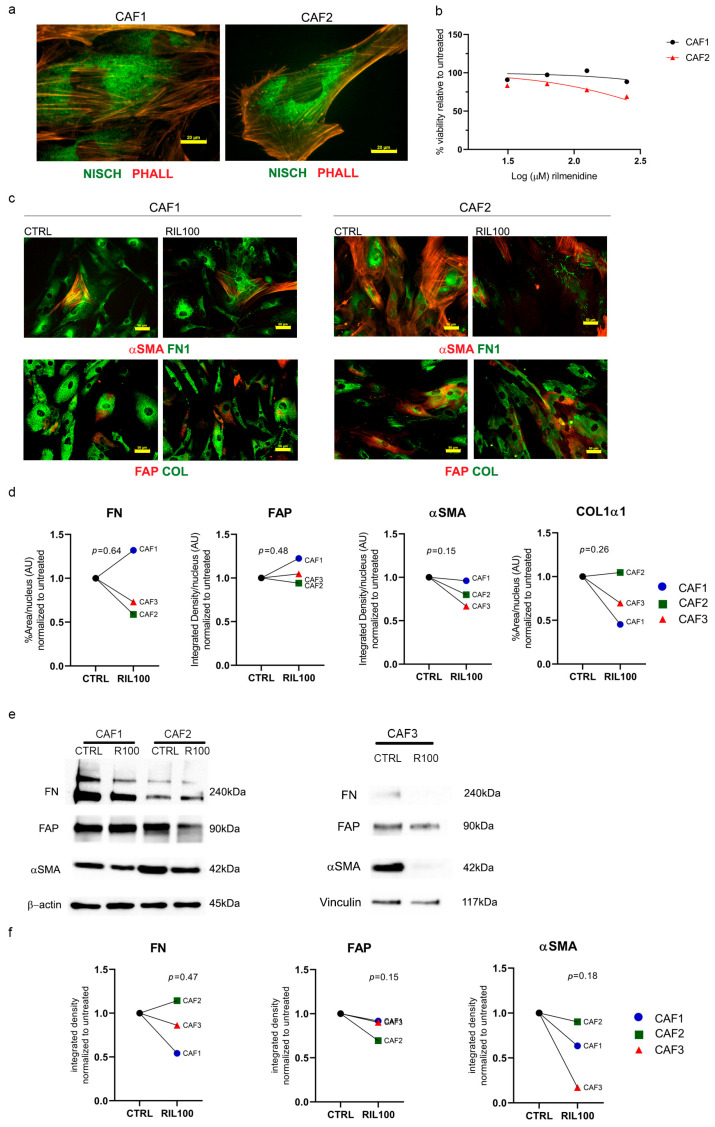
Rilmenidine affects CAF phenotype (**a**) NISCH expression (green) in CAFs isolated from two patient tissues, phalloidin red, scale bar 20 μm; (**b**) percent viability of CAFs after 72 h of treatment with increasing concentrations of rilmenidine, determined by MTT assay, presented as log(inhibitor) vs. normalized response; (**c**) immunofluorescence staining of CAF markers α-SMA (red), FAP (red), collagen I (green) and fibronectin (green) after 72 h of rilmenidine treatment in CAF1 (left panel) and CAF2 (right panel) cells, scale bar 50 μm; (**d**) quantification of the immunofluorescence staining with Image J, expressed as percent area per nucleus for FN1 and COL1 and as integrated density per nucleus for α-SMA and FAP, for three CAF cell lines, normalized to the untreated condition. *p*-values from paired *t*-test; (**e**) expression of α-SMA, FAP and fibronectin in CAF1, CAF2 and CAF3 cell lysates, after 72 h of rilmenidine treatment; (**f**) quantification of western blots with Image J presented as integrated density of the select protein divided by the integrated density of the housekeeping protein normalized to the untreated condition. *p*-values from paired *t*-test.

NISCH was also expressed in the stromal compartment of PDAC and in CAFs isolated from PDAC tumor tissue (Figure 2f and Figure 6a); therefore, we examined the effects of rilmenidine on CAF cell fitness. In the MTT assay with up to 600 μM rilmenidine treatment for 72 h, no effects on CAFs viability were observed (Figure 6b).

**Figure 7:**
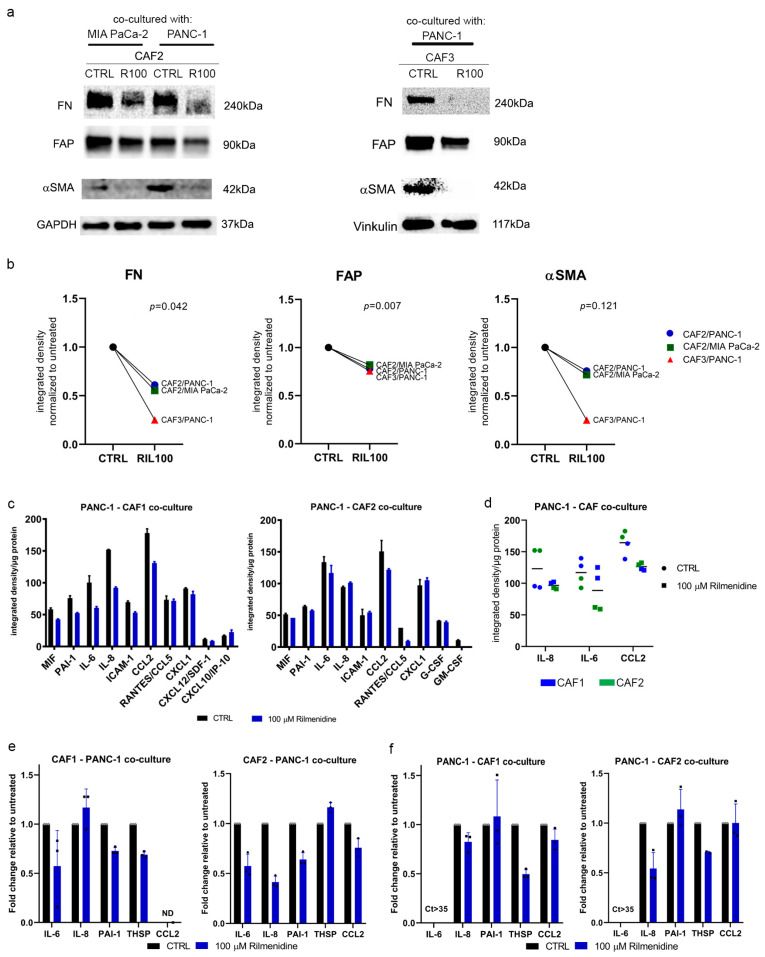
Rilmenidine affects CAF phenotype in co-culture and alters production of pro-tumorigenic cytokines. (**a**) Expression of α-SMA, FAP and fibronectin in CAF2 from co-culture with either PANC-1 or MIA PaCa-2 cells and CAF3 cells from co-culture with PANC-1 cells, untreated or treated with 100 μM rilmenidine; (**b**) Quantification of western blots with Image J presented as integrated density of the select protein divided by the integrated density of the housekeeping protein normalized to the untreated condition. *p*-values from paired *t*-test. (**c**) Quantification of the integrated density of signal from the dot blot analysis of conditioned media from PANC-1—CAF1 and PANC-1—CAF2 co-cultures treated with 100 μM rilmenidine; (**d**) Select iCAF cytokine profile changes from dot blot analysis of conditioned media from PANC-1—CAF1 and PANC-1-CAF2 co-cultures treated with 100 μM rilmenidine. Mean, from two biological experiments in technical duplicate; (**e**) mRNA expression of select cytokines in CAF1 and CAF2 cells from co-cultures with PANC-1 cells treated with rilmenidine; (**f**) mRNA expression of select cytokines in PANC-1 cells from co-cultures with CAF1 or CAF2 cells, expressed as fold change to untreated control. The graphs show the mean of technical triplicates.

Next, we examined the expression of CAF markers after treatment with 100 μM rilmenidine—FAP, αSMA, fibronectin (FN1), and collagen I alpha 1 (COL1α1). Immunofluorescence staining (Figure 6c and Supplementary Figure S5, quantification in 6d) showed a varied extent of decrease in the expression of αSMA and deposition of FN and COL1, which were cell line dependent, while FAP expression remained unchanged. Immunoblotting confirmed a trend in the decrease of αSMA expression in all three cell lines (Figure 6e,f), while in the CAF2 line, there was also a reduction in FAP expression after treatment. As secreted growth factors in CAF-cancer cell co-cultures support cell growth [43], we examined whether the observed changes in CAFs hold true in the presence of cancer cells. We cultured CAF2 or CAF3 cells in TW co-culture with PANC-1 or MIA PaCa-2 cells and examined CAF marker expression by immunoblotting (Figure 7a,b). We observed a decrease in the expression of FN in the presence of rilmenidine compared to the control in the co-cultured cells, as well as a trend in a decrease in the expression of αSMA.

CAFs constitute a barrier for chemotherapy and targeted therapy and can produce signaling molecules that can facilitate tumor progression and metastasis [43]. The tumor microenvironment communicates with cancer cells via a variety of secreted growth factors and cytokines. Cancer cell-derived IL-1 induces a signaling cascade in CAFs that leads to JAK/STAT activation and promotes the pro-inflammatory phenotype [44] and in co-culture with PDAC cancer cells, the CAF population shifts toward the iCAF phenotype [45]. To screen for changes in cytokines and chemokines under rilmenidine treatment, we examined the medium of CAF-cancer cell co-cultures with a dot blot array. While the basal cytokine profiles of the conditioned medium of CAF1 and CAF2 cells co-cultured with the PANC-1 cancer cell line differed, as well as their response to rilmenidine (Figure 7c), we observed a common trend in the change in the cytokines associated with the pro-inflammatory iCAF phenotype, CCL2, IL-6, and IL-8 [8,46] (Figure 7d). ELISA assays on CAF2-cancer cell co-culture media confirmed decreased IL-6 and IL-8 secretion in co-culture media upon rilmenidine treatment (Supplementary Figure S6a,b). PAI-1 levels were higher in CAF-PANC-1 co-cultures than in CAFs alone; however, rilmenidine treatment did not alter its expression (Supplementary Figure S6c).

Next, we examined the mRNA expression of cytokines and components of the fibrotic signature in CAFs and PANC-1 cells from the co-cultures. We found a decrease in *IL6* and *PAI-1* mRNA levels in both CAF1 and CAF2 cells from co-cultures after 72 h of treatment with 100 μM rilmenidine (Figure 7e) and a decrease in *IL8* mRNA levels in the CAF2 cell line. *IL8* decreased only in PANC-1 cells co-cultured with CAF2, while PANC-1 cells co-cultured with CAF1 showed a notable decrease in THSP mRNA levels (Figure 7f).

Taken together, our results imply that although there was variability in the response between CAFs from different patients, rilmenidine had an effect on the phenotype of CAFs from the TME and that it had the potential to reduce communication between CAFs and cancer cells, which is a crucial process in PDAC progression.

## 4. Discussion

Although the five-year survival rate of patients with PDAC has surpassed 10% in the last decade, the increase in survival is still modest, and the incidence of the disease is increasing [47]. PDAC is fatal once spread beyond the primary site. With limited treatment options, research has focused on discovering better therapeutic targets, especially for the metastatic disease, and biomarkers that will enable earlier detection. In PDAC patients who have undergone surgical resection, recurrence occurs in the next 12 to 24 months [48]. This creates a window of opportunity for the application of potential antimetastatic therapies. Metastatic spread is a complex cascade that includes cell detachment, migration, local tissue invasion, intravasation into the vasculature or lymphatics, survival in the circulation, extravasation, attachment, and adaptation to the new microenvironment, growth of micrometastases, and the establishment of macrometastases [49]. Targeting these processes may help limit the PDAC progression.

NISCH was reported to be a tumor suppressor in breast and ovarian tumors, with decreased NISCH expression promoting cell migration and modulating cell-cell and cell-ECM adhesion [15]. As control of adhesion and migration are prerequisites for cancer cell invasion and spread [50], NISCH is a potentially attractive antimetastatic drug target. Three clinically approved antihypertensive drugs bind to IRAS-I1/NISCH receptor: rilmenidine, moxonidine, and clonidine [17]. We investigated whether these drugs have the potential to be repurposed in PDAC.

We previously examined NISCH expression in datasets from public repositories in various tumor types and reported that it was expressed to a greater or lesser extent in all the examined tumor tissues, most often decreased compared to their healthy counterparts [16]. In this study, we found that NISCH was expressed in pancreatic cancer, but there was no significant difference in its expression between the tumor and healthy pancreatic tissue. However, based on the expression level in tumors, *NISCH* was a positive prognostic marker for both the progression-free and overall survival of patients with PDAC. The possible context-dependent role where *NISCH* might not be involved in PDAC tumor initiation (hence no difference in expression between tumor and normal) but influences tumor progression, metastasis or treatment resistance can translate into an association with survival. We found no difference in *NISCH* expression or its prognostic value between the sexes in PDAC. Notably, we found that NISCH was expressed in both the epithelial and stromal compartments of PDAC tissue and in cancer cells and CAFs in vitro. As the stroma plays a significant role in PDAC tumor progression and response to therapy [51], NISCH expression in both compartments implies that systemically administered NISCH agonists may have diverse effects. As we previously reported [16], NISCH could be observed in nuclei of cells in PDAC tissues. NISCH does not contain classic DNA-binding motifs; however, as it has been described as a molecular scaffold [52], it could potentially be part of the nuclear matrix in cancer cells and scaffold nuclear signaling processes. This observation warrants further exploration.

We found that rilmenidine was the most potent drug in vitro in the MTT assay, which was in agreement with rilmenidine having the highest selectivity for binding to NISCH [18]. Although rilmenidine ultimately induced cancer cell apoptosis, the concentrations needed to induce this effect were much higher for PDAC cell lines than those previously reported by our group and others, for melanoma cells [31] and K562 leukemia cells [53]. We observed differences in sensitivity to rilmenidine among the tested cell lines, irrespective of their doubling time or the amount of NISCH expressed, modeling heterogeneity. Transcriptome analysis of the most sensitive cell line, MIA PaCa-2, treated with rilmenidine showed enrichment of pathways associated with cell adhesion and migration, as expected from the NISCH function.

We confirmed in in vitro assays that rilmenidine treatment attenuated and, at a high dose, prevented cancer cell attachment on various ECM substrates, and that it inhibited cell migration. This was in agreement with the findings that NISCH regulated focal adhesion and invadopodia formation [54]. We also showed that, at least in PANC-1 cells in conditions of collective migration, rilmenidine decreases the level of active Rac1. However, in the cell invasion assay, rilmenidine only modestly impaired invasion irrespective of the matrix coating, apart from the BxPC-3 cell line, where inhibition was significant. Mesenchymal (dependent on adhesion and MMP activity) and amoeboid (independent of MMP activity) modes of movement through matrices are dictated by both intrinsic cell characteristics and the microenvironment through which they invade [55], and cancer cells can move through matrices even without any ECM adhesion [56]. Given that PDAC cells show phenotypic plasticity when it comes to the type of migration and can migrate both dependently and independently of MMP secretion [42], we speculate that the effects of rilmenidine may be dispensable in quasimesenchymal cell lines PANC-1 and MIA PaCa-2 when they move through matrices without ECM adhesion. Examining gelatinolytic activity, we found that BxPC-3 cells expressed higher amounts of collagenases than the other two lines, and that rilmenidine slightly decreased their activity. Most recently, it has been shown that NISCH harbors redox-regulatory cysteine (Cys185), where S-glutathionylation under conditions of oxidative stress prevents NISCH interaction with Rac1 and PAK1, thus reducing its antimigratory role [57]. It remains to be examined whether the status of NISCH S-glutathionylation in cancer cells influences the effects of rilmenidine on migration.

Ultimately, to ascertain the antimetastatic potential of rilmenidine, we examined its effect on metastasis in the zebrafish model. Tg(*fli1*:EGFP) zebrafish, in which the promoter of the endothelial marker *fli1* drives the expression of enhanced green fluorescent protein (EGFP) in blood vessels, enables easy visualization and quantification of metastatic cancer cell seeding [58]. In the PANC-1 tumor xenograft zebrafish model, all embryos developed metastasis, with an average number of 6. Treatment with 50 μM rilmenidine significantly reduced the number of metastases per embryo, and at 100 μM, more than 70% of the embryos had no detectable metastases. In the zebrafish model, tumor cells that exit the injection site circulate through the vasculature, arrest in the vessels, and adhere to endothelial surfaces [59]. Strong adhesions mediate stable endothelium bonds, which are a prerequisite for extravasation [60]. The observed lower metastatic burden in zebrafish, together with the in vitro assays in human PDAC cell lines, implies that rilmenidine is affecting metastatic colonization by decreasing cell adhesion. However, given that the zebrafish model is not representative of the PDAC TME, lacking CAFs and the adaptive immune response, it remains to be tested whether rilmenidine may have antimetastatic properties in the appropriate mouse PDAC models of metastasis.

As the main feature of PDAC is the prominent TME, it was imperative to examine the effects of treatment on as many cell types as possible that are present in the tumor. Cancer cells and CAFs undergo dynamic crosstalk through direct cell-to-cell contact and the secretion of growth factors, cytokines, and extracellular vesicles. This bidirectional communication can have both tumor-promoting and tumor-suppressive roles in PDAC development [61]. CAFs can promote the migration and invasion of cancer cells, as well as drug resistance [62], and can create a pro-inflammatory microenvironment [8]. Several distinct CAF phenotypes have been identified, of which the three main types—myofibroblasts (myCAFs), inflammatory CAFs (iCAFs), and antigen-presenting CAFs (apCAFs)—are the most well-described. MyCAFs are mostly located adjacent to neoplastic cells and are characterized by high expression of αSMA and lower expression of FAP, iCAFs express FAP and secrete cytokines such as IL-6, which support the growth of cancer cells [8], while apCAFs express the MHC II complex and CD74 and can present antigens to T cells in vitro [63]. CAF phenotypes are not fixed, and subtypes can transition between subsets [43]. It has been suggested that FAP^+^ and αSMA^+^ CAFs are distinct subpopulations with distinct functions, with αSMA positivity correlating with increased overall survival and FAP positivity correlating with significantly decreased overall survival of PDAC patients [64]. We isolated mixed populations of CAFs from three patients and examined the effects of rilmenidine on both monoculture and indirect cancer cell-CAF co-cultures. NISCH was expressed in CAFs, but even high doses of rilmenidine (up to 600 μM) did not affect their viability. While we were not able to perform the whole battery of experiments on all three established cell lines due to the cell senescence, in all three CAF lines cultured alone, rilmenidine treatment showed a trend in a decrease of both αSMA and FAP protein expression. Treatment with rilmenidine in the co-culture conditions did not change αSMA expression further, but decreased FAP expression in all three CAF cell lines. FAP has been shown to be a persistent activator of fibroblastic STAT3, where it triggers induction of a CAF subset with an inflammatory phenotype marked by CCL2 upregulation [65]. Indeed, in the CAF1 and CAF2 co-culture with PANC-1 cells, we were able to detect CCL2 in the medium, and its decrease in the rilmenidine-treated condition.

These findings are important because they imply that rilmenidine may contribute to the normalization of the tumor stroma. Rilmenidine treatment decreased the production of IL-6, IL-8, and CCL-2 in co-cultures. These results are promising, as IL-6 plays an important role in PDAC progression [66]. IL-6 secreted by CAFs stimulates the activation of STAT3 signaling in tumor cells and promotes invasion [67]. IL-6/STAT3 signaling also contributes to tissue fibrosis [68], and the inhibition of IL-6 signaling has shown promising results in the inhibition of pancreatic tumor progression [69]. We also observed a decrease in PAI-1 production in co-cultures, as well as a decrease in the transcription of *THSP*, another fibrosis marker, in both CAFs and cancer cells. Taken together, our findings imply that rilmenidine treatment may have positive effects on the tumor stroma by attenuating the CAF pro-tumorigenic phenotype, decreasing the fibrotic signature, and disrupting cancer cell-CAF communication. A common node for IL-6, IL-8, CCL-2 and PAI changes is the JAK/STAT3 signaling pathway, and given that STAT3 activation has a role in shaping both PDAC cell [45] and CAF phenotypes [44], it remains to be elucidated whether and at which level rilmenidine treatment and NISCH expression impact this pathway. As any therapy that alters the stromal composition or communication can have beneficial or harmful effects depending on the timing of administration, the stromal content and the PDAC subtype, effects of rilmenidine on STAT3 status should further be examined in vivo.

One limitation of our study was the use of relatively high doses of rilmenidine. Specifically, after an oral dose of 3 mg in patients, peak plasma concentrations of rilmenidine reach about 10 ng/mL [70], which falls within the nanomolar range, whereas in functional assays in vitro, we used concentrations from 50 to 300 μM. In vitro studies often rely on supra-physiological doses to produce clear and rapid cellular responses that may not be detectable under normal physiological conditions within typical laboratory timeframes. A similar example is the repurposing of metformin, where achievable plasma levels were far lower than the concentrations required in vitro to generate biological effects in cancer cells [71]. Have metformin not been shown to promote survival in cancer patients with diabetes, in vitro studies in DMEM would concluded that metformin has no effect on cancer cells in clinically achievable concentrations [72]. In our study, 100 μM rilmenidine showed no toxicity in an in vivo zebrafish model, while the number of metastases per embryo decreased significantly at 50 μM. Higher doses of rilmenidine were also used in other model organisms, for example, in *Caenorhabditis elegans*, where up to 400 μM rilmenidine was tested, and a dose of 200 μM was shown to significantly extend the lifespan of animals [73]. Of importance, in a mouse model of amyotrophic lateral sclerosis, a dose of 10 mg/kg was used without toxicity [74], a dose that for a 70 kg human corresponds to 57 mg. Should rilmenidine prove to be effective for treatment in mouse models of PDAC, the next step would involve optimizing its dosing for this new indication. In this context, several strategies could be explored, including liposomal formulations, as has been done with the antihypertensive drug losartan [75,76], which enable the use of higher doses. Additionally, a novel analog of rilmenidine with more potent effects at smaller doses has recently been patented (PCT/US2025/043771).

We present results only on the mechanistic level in vitro, and our findings remain to be corroborated in appropriate PDAC mouse models in both primary tumor and metastatic spread settings. However, our study lays the foundation for further investigation of the repurposing potential of the antihypertensive rilmenidine in the treatment of PDAC, as we show that it targets at least two aspects of PDAC progression: cancer cell dissemination and cancer cell-stroma communication. While repurposing rilmenidine would provide faster translation to the clinic, the design and development of novel NISCH agonists may also be of interest, as we showed that NISCH is expressed in both tumor and stromal compartments and its agonization may have a dual effect.

## 5. Conclusions

Nischarin is expressed in both epithelial and stromal compartments of PDAC. Its agonist rilmenidine has an impact on both cancer cells and CAFs in vitro and their communication, with antimetastatic effects in the tumor xenograft zebrafish model. We show that rilmenidine is a promising candidate for further preclinical investigation for repurposing in PDAC.

## Data Availability

The data supporting the findings of this study are available from the corresponding author upon reasonable request. The public data of NISCH mRNA and protein expression levels in PDAC and normal pancreatic tissues in this study were obtained from the public repositories https://portal.gdc.cancer.gov, https://www.cbioportal.org/, http://gepia2.cancer-pku.cn/, https://ualcan.path.uab.edu/ and www.proteinatlas.org/ (all accessed on 30 July 2024). Raw sequencing data of MiaPaca2 cell line mRNA transcriptome, treated for 24 h or 48 h in vitro with 100 μM rilmenidine can be found at https://zenodo.org/records/6920520 and https://zenodo.org/records/6948536 (accessed on 30 July 2024), respectively.

## Supplementary Materials

The following supporting information can be downloaded at: https://www.mdpi.com/article/10.3390/cells15111032/s1, Supplementary Table S1: List of primary antibodies used in immunoblotting (western blot—WB), immunohistochemistry (IHC) or immunofluorescence staining (IF); Supplementary Table S2: List of assays performed on CAF cell lines; Supplementary Table S3: Sequences of the primers used for qRT-PCR analysis; Supplementary Table S4: Lethal and teratogenic effects observed in zebrafish (*Danio rerio*) embryos at different hours post fertilization (hpf); Supplementary Table S5: Effect of nischarin agonists on viability of the pancreatic cancer cell line panel; Supplementary Figure S1: NISCH expression in PDAC; Supplementary Figure S2: NISCH agonist rilmenidine reduces cell adhesion and migration in vitro; Supplementary Figure S3: Annexin V+/PI- cells in the population of untreated and cells co-treated at the time of seeding with 100 or 300 μM rilmenidine assayed after 48 h. Supplementary Figure S4: Rilmenidine effects on the gelatinolytic activity of MMP-2 and MMP-9 in PDAC cells in vitro; Supplementary Figure S5: Rilmenidine affects the CAF phenotype; Supplementary Figure S6: Rilmenidine impacts the level of cytokine production in co-cultures and patient tissues; Supplementary Figure S7: Downregulation of NISCH expression increases directional migration of MIA PaCa-2 cells and decreases their response to rilmenidine. Reference cited in Supplementary Materials [77].

## Abbreviations

The following abbreviations are used in this manuscript:

PDAC	Pancreatic ductal adenocarcinoma
TME	Tumor microenvironment
NISCH	Nischarin
Rac1	Rac Family Small GTPase 1
PAK1	p21 (RAC1) Activated Kinase 1
LIMK1	LIM domain kinase 1
TCGA	The Cancer Genome Atlas
GEO	Gene Expression Omnibus
HRP	Horseradish Peroxidase
GO	Gene Ontology
EGFP	Enhanced Green Fluorescent Protein
CAF	Cancer-Associated Fibroblasts
apCAFs	Antigen-presenting cancer-associated fibroblasts
iCAFs	Inflammatory cancer-associated fibroblasts
myCAFs	Myofibroblasts
FAP	Fibroblast Activation Protein
αSMA	Alpha-Smooth Muscle Actin
TW	Transwell
MMPs	Matrix metalloproteinases
COL1α1	collagen type I alpha 1
FN1	Fibronectin 1
IL-6	Interleukin 6
IL-8	Interleukin 8
PAI-1	Plasminogen Activator Inhibitor-1
THSP	Thrombospondin
