# Supplementary material for: Repurposing Rilmenidine as a Potential Antimetastatic Therapy Targeting Nischarin in Pancreatic Ductal Adenocarcinoma
Source: Cells. 2026 Jun 3;15(11):1032. doi: 10.3390/cells15111032 (PMC13256129; doi:10.3390/cells15111032)
Supplement: Supplementary file 1 [file cells-15-01032-s001.zip › cells-4348447-supplementary.pdf]

Supplementary Materials

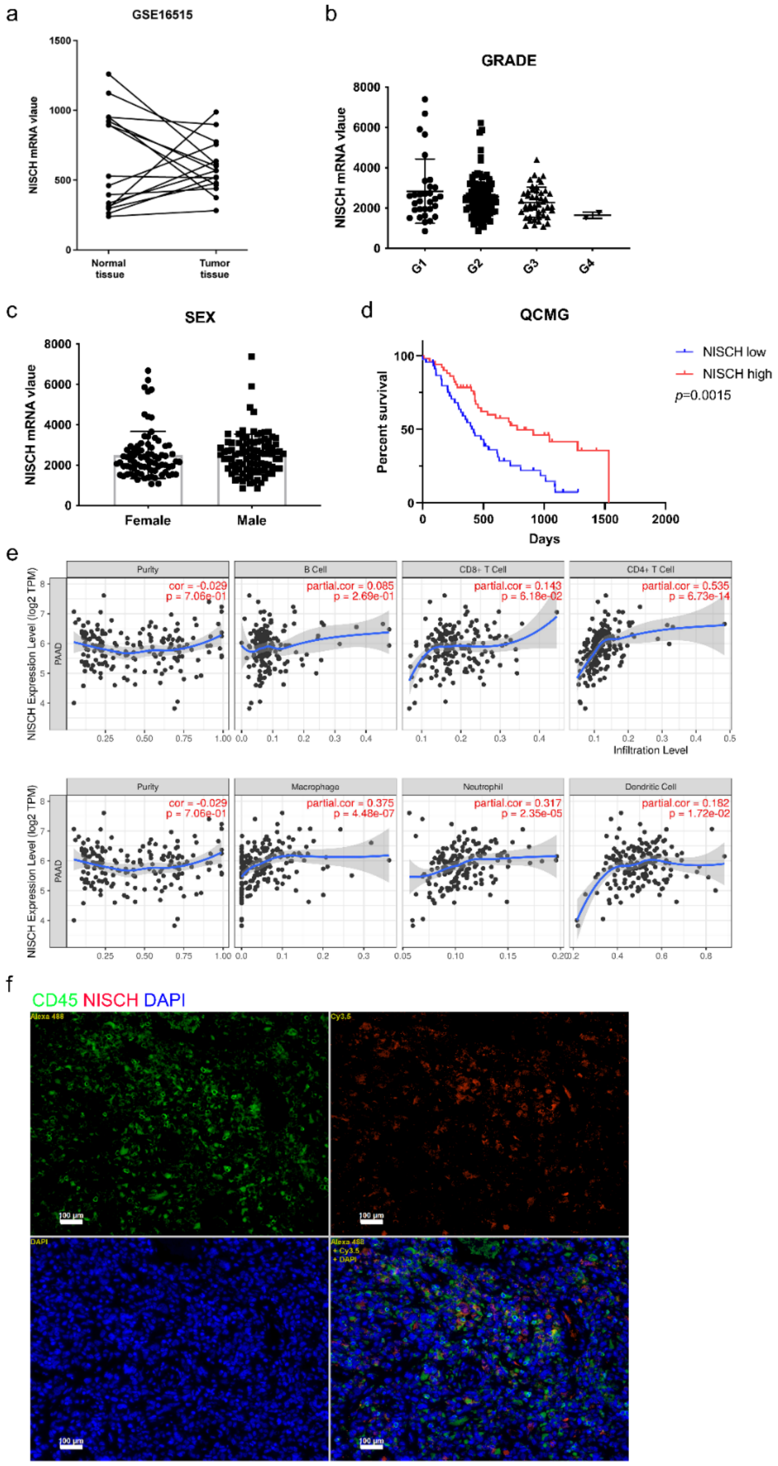

Figure S1. NISCH expression in PDAC. **a**) *NISCH* mRNA expression in the paired tumor and adjacent PDAC tissue samples in the GSE16515 cohort; **b**) *NISCH* mRNA expression by grade in the TCGA PAAD cohort **c**) *NISCH* mRNA expression by patient sex in the TCGA PAAD cohort; **d**) Kaplan-Meier plot for the overall survival of PDAC patients in the Bailey data set cohort divided into two groups using the best cut-off value for NISCH. $p = 0.0015$ by Mantel-Cox test **e**) Correlation of *NISCH* expression with immune infiltration level in PDAC, shown are purity-corrected partial Spearman's rho value and statistical significance (Li et al. 2017) **f**) Expression of NISCH (red) and CD45 (green) in PDAC liver metastasis sample from the NBP2-78128 microarray, nuclei DAPI blue, scale bar 100 μm.

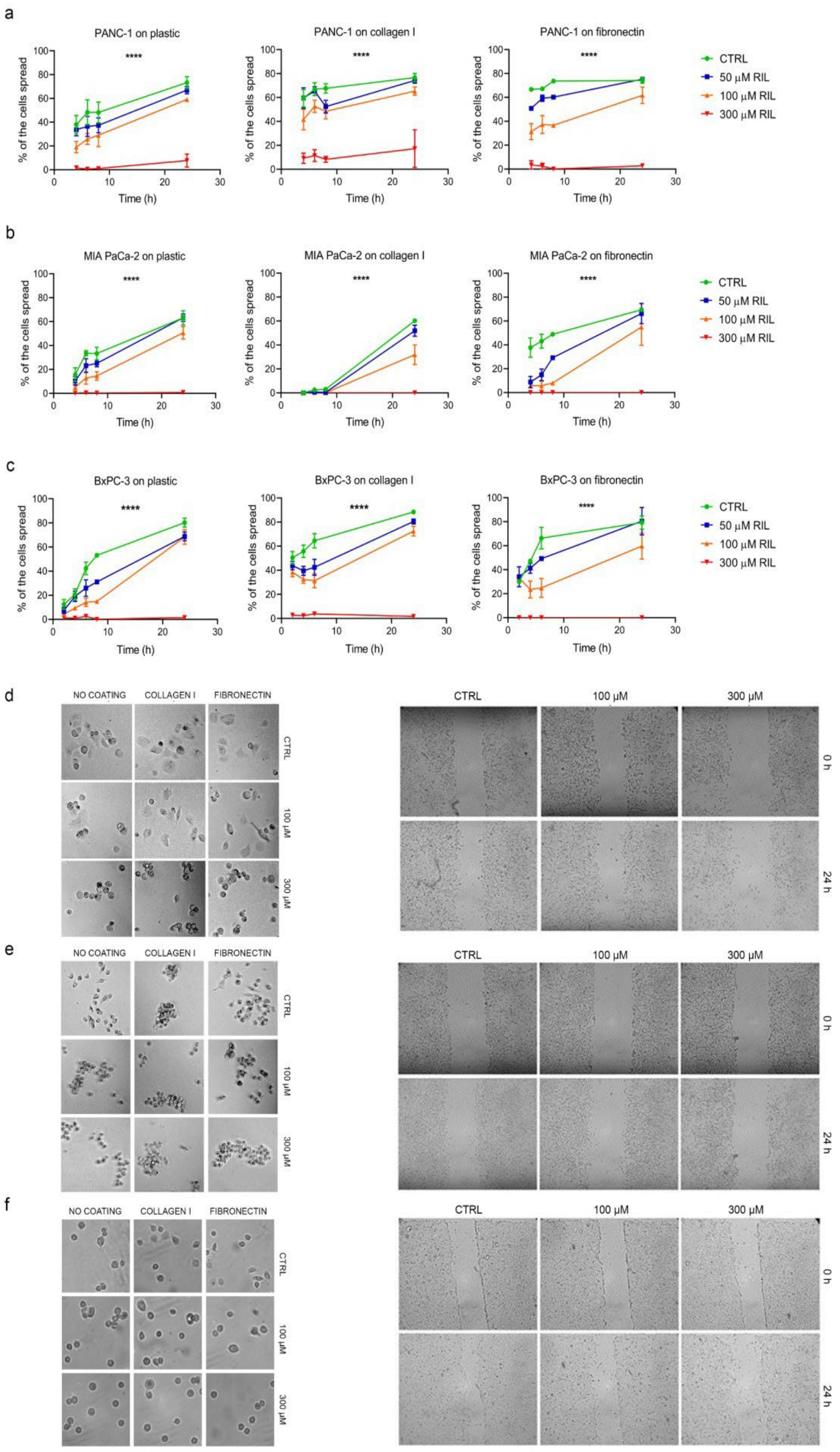

Figure S2. NISCH agonist rilmenidine reduces cell adhesion and migration *in vitro*. **a**) Quantification of PANC-1, **b**) MIA PaCa-2 and **c**) BxPC-3 cell spreading over time, in presence of increasing concentrations of rilmenidine on tissue culture plastic, collagen I and fibronectin coated surfaces. Mean ± SD. Two-way ANOVA (Sidak's multiple comparisons test), n = 3; *$p < 0.05$, **$p < 0.01$, ***$p < 0.001$, ****$p < 0.0001$. **d**) Representative images of PANC-1, **e**) MIA PaCa-2 and **f**) BxPC-3 cell spreading (left panel) and wound closure (right panel) for after 24 hours of rilmenidine treatment. Magnification: 10×.

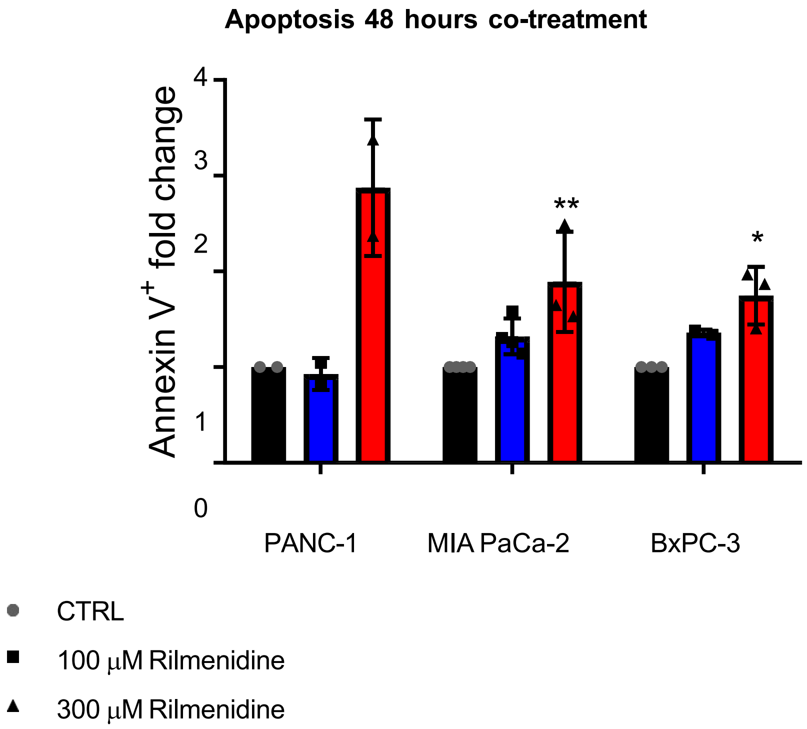

Figure S3. Annexin V+/ PI- cells in the population of untreated and cells co-treated at the time of seeding with 100 or 300 μM rilmenidine assayed after 48 hours. Results are shown as fold change of Annexin V+/ PI- cells compared to the untreated control. All data are shown as mean ± SD of biological duplicates or biological triplicates; *$p < 0.05$, **$p < 0.01$, two-way ANOVA (Sidak's multiple comparisons test) determined only for cell lines and treatments with n=3.

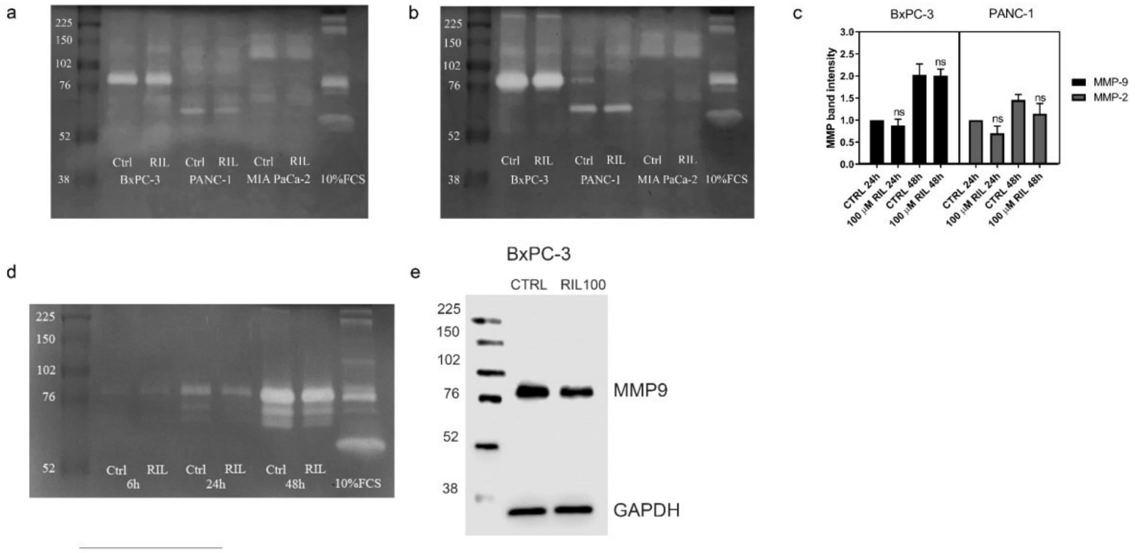

Figure S4. Rilmenidine effects on the gelatinolytic activity of MMP-2 and MMP-9 in PDAC cells *in vitro*. **a**) Representative gelatin zymograms of 24 h or **b**) 48 h conditioned medium of PANC-1, MIA PaCa-2, and BxPC-3 cells treated with 100 μM rilmenidine **c**) Quantification of the intensities of cleared bands analyzed with ImageJ and normalized to untreated. Mean ± SD, Two-way ANOVA (Sidak's multiple comparisons test), n = 3; (compared to the untreated control). **d**) Gelatin zymography of BxPC-3 cell lysates 6 h, 24 h, and 48 h after the treatment with 100 μM rilmenidine. **e**) MMP-9 expression in BxPC-3 cell lysates after 48 h of rilmenidine treatment.

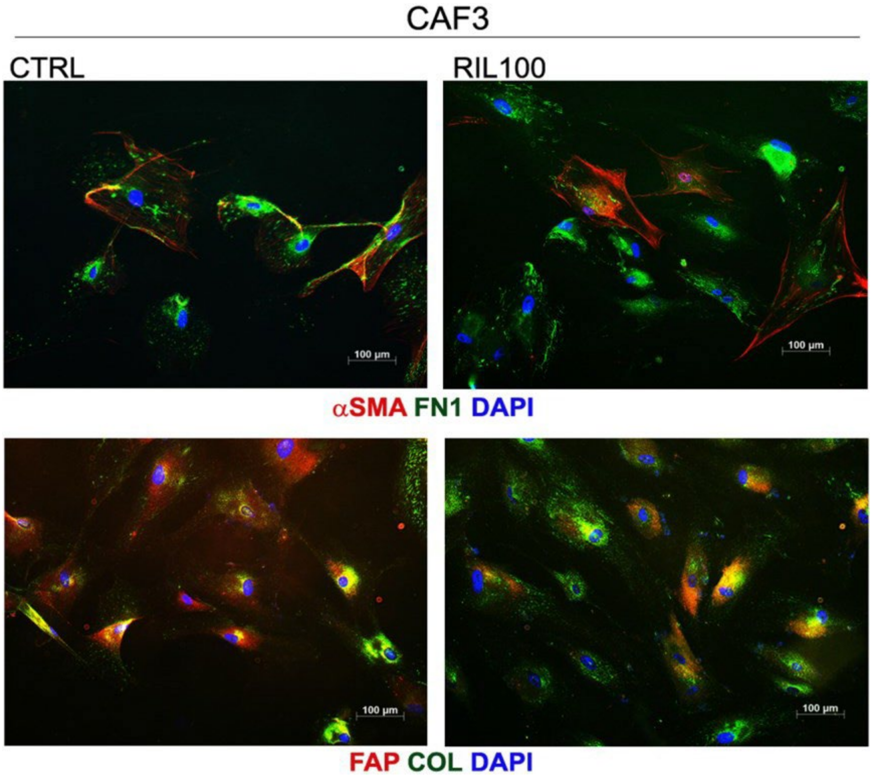

Figure S5. Rilmenidine affects the CAF phenotype. Immunofluorescence staining of CAF protein markers α-SMA (red), FAP (red), collagen I (green) and fibronectin (green) after 72 hours of rilmenidine treatment in CAF3 cells, scale bar 100 µm (left panel) and quantification of integrated density of staining per nucleus, normalized to untreated controls (right panel).

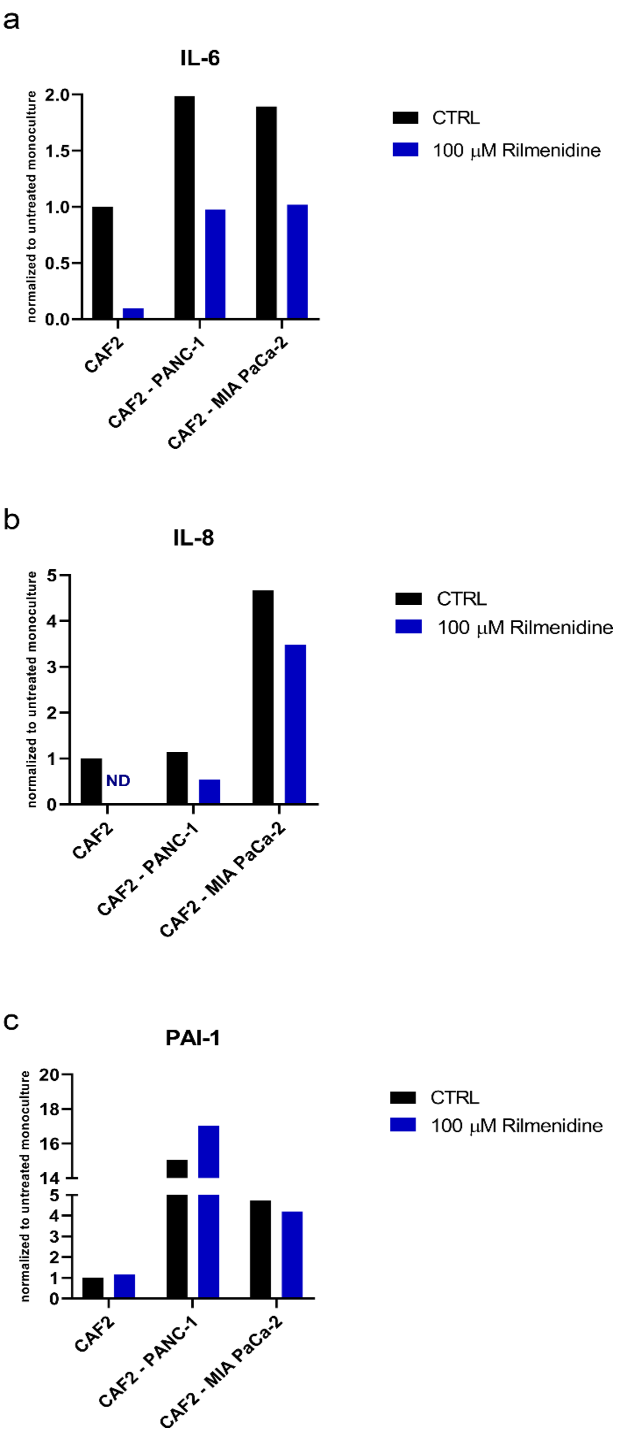

Figure S6. Rilmenidine impacts the level of cytokine production in co-cultures and patient tissues. Levels of **a**) interleukin 6, **b**) interleukin 8 and **c**) plasminogen activator inhibitor 1 in culture media of CAFs and pancreatic cancer cells co-cultures after treatment with 100 μM rilmenidine.

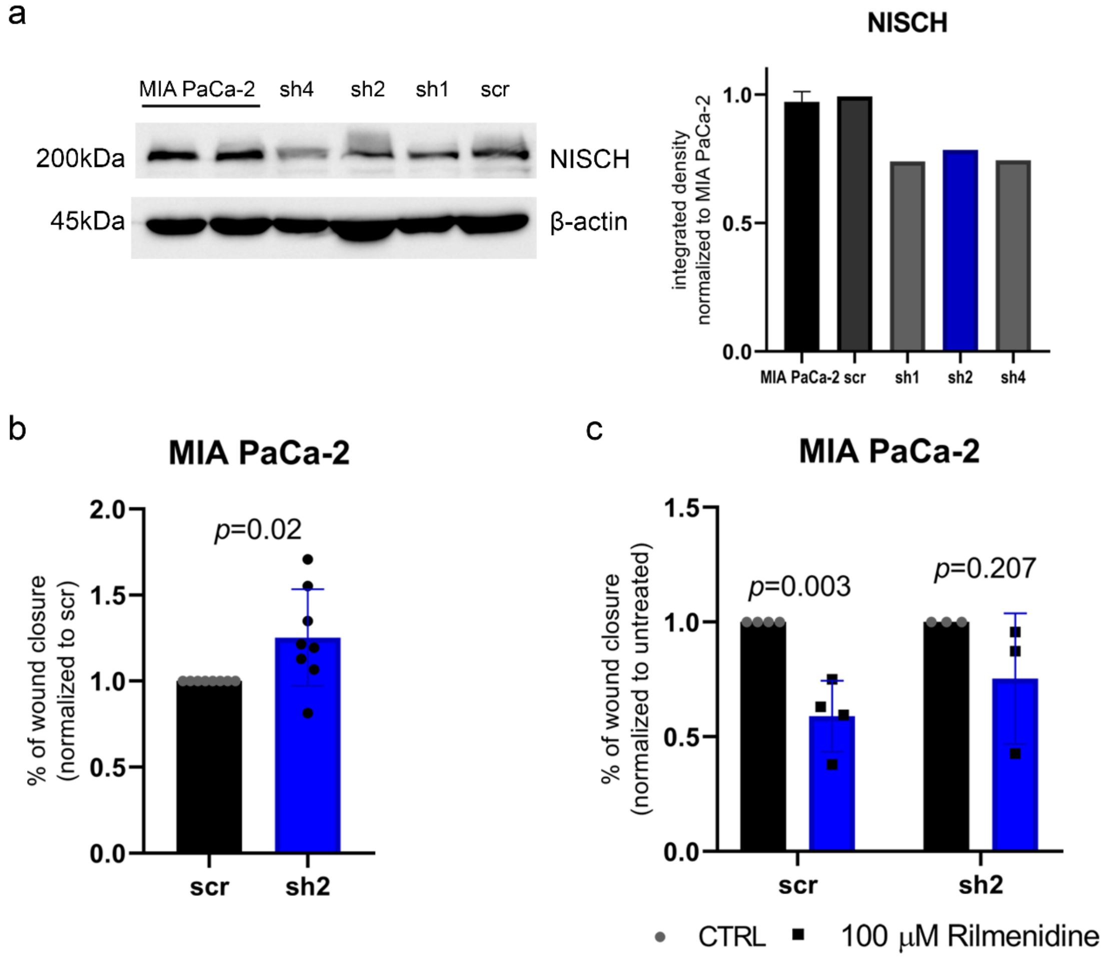

Figure S7. Downregulation of NISCH expression increases directional migration of MIA PaCa-2 cells and decreases their response to rilmenidine. **a**) Immunobloting of NISCH in parental MIA PaCa-2 cell line and stabile shNISCH KD MIA PaCa-2 and sh scramble cell lines, left panel. Quantification with Image J presented as integrated density of NISCH divided by the integrated density of β-actin normalized to the parental cell line, right panel. **b**) Quantification of sh scramble and shNISCH-2 MIA PaCa-2 cell migration 24 hours after wounding in the wound healing assay, normalized to scramble. **c**) Quantification of the migration in the wound healing assay after 24 hours of treatment with rilmenidine of sh scramble and shNISCH-2 MIA PaCa-2 cells. Results are represented as % of wound closure normalized to untreated control for each cell line. Mean ± SD, unpaired t-test.

References

Li, T., Fan, J., Wang, B., Traugh, N., Chen, Q., Liu, J.S., Li, B. & Liu, X.S., 2017, 'TIMER: A web server for comprehensive analysis of tumor-infiltrating immune cells', *Cancer Research*, 77(21), e108–e110.

Table S1. List of primary antibodies used in immunoblotting (western blot – WB), immunohistochemistry (IHC) or immunofluorescence staining (IF).

Primary antibody	***Catalog number***	***Manufacturer***	***Dilution***		
			**WB**	**IF**	**IHC**
Anti-NISCH	558262	Biosciences	/	1:150	1:300
Anti-NISCH	HPA023189	Sigma-Aldrich	/	/	1:30
Anti-α-SMA	19245	Cell signaling technology	1:1000	1:150	1:150
Anti-CD45	CD45-2B11	Invitrogen	/	/	1:100
Anti-NISCH	85124	Cell signaling technology	1:1000	/	/
Anti-fibronectin 1	610077	BD Biosciences	1:10000	1:100	/
Anti-FAP	66562	Cell signaling technology	1:1000	1:50	1:50
Anti-β-actin	3700	Cell signaling technology	1:2000	/	/
Anti-Gapdh	sc-365062	Santa Cruz Biotechnology	1:1000	/	/
Anti-MMP-9	MAB936	R&D Systems	1:500	/	/
Anti-Vinculin	FAK100; Part No. 90227	Merck Millipore	1:1000	/	/
Anti- COL1α1	MAB6220	R&D Systems	/	1:50	/
Cytokeratin19	MAB35061	R&D Systems			
E-cadherin	610181	BD Biosciences	/		/

Table S2. List of assays performed on CAF cell lines.

Experiment	*CAF1*	*CAF2*	*CAF3*
ICC NISCH	+	+	-
MTT assay	+	+	-
ICC fibroblast markers	+	+	+
Monoculture WB	+	+	+
Co-culture WB	-	+	+
Co-culture qPCR	+	+	-
Dot blot	+	+	-

Table S3. Sequences of the primers used for qRT-PCR analysis.

Gene	*Forward primer*	*Reverse primer*
**ACTA2**	AGGGGGTGATGGTGGGAATG	GCCCATCAGGCAACTCGTAAC
**FAP**	GGAAGTGCCTGTTCCAGCAATG	TGTCTGCCAGTCTTCCCTGAAG
**COL1A1**	GAGGGCCAAGACGAAGACATC	CAGATCACGTCATCGCACAAC
**FN1**	CGGTGGCTGTCAGTCAAAG	AAACCTCGGCTTCCTCCATAA
**IL6**	ACTCACCTCTTCAGAACGAATTG	CCATCTTTGGAAGGTTCAGGTTG
**IL8**	GAGAGTGATTGAGAGTGGACCAC	CACAACCCTCTGCACCCAGTTT
**PAI1**	CTCATCAGCCACTGGAAAGGCA	GACTCGTGAAGTCAGCCTGAAAC
**THSP**	GCTGGAAATGTGGTGCTTGTCC	CTCCATTGTGGTTGAAGCAGGC
**CCL2**	AGAATCACCAGCAGCAAGTGTCC	TCCTGAACCCACTTCTGCTTGG
**ACTB**	TCTGGCACCACACCTTCTAC	GATAGCACAGCCTGGATAGC
**GAPDH**	GACAGTCAGCCGCATCTTCT	GCGCCCAATACGACCAAATC

Table S4. Lethal and teratogenic effects observed in zebrafish (*Danio rerio*) embryos at different hours post fertilization (hpf).

Category	*Toxicological parameters*	*Exposure time (hpf)*				
		**24**	**48**	**72**	**96**	**120**
Lethal effect	Coagulated eggs[a]	●	●	●	●	●
	Lack of the heart beating	●	●	●	●	●
	Non-detachment of the tail	●	●	●	●	●
	Lack of somite formation	●	●	●	●	●
Teratogenic effect	Malformation of head	●	●	●	●	●
	Malformation of eyes[b]	●	●	●	●	●
	Malformation of sacculi/otoliths[c]	●	●	●	●	●
	Malformation of chorda	●	●	●	●	●
	Malformation of tail[d]	●	●	●	●	●
	Scoliosis	●	●	●	●	●
	Yolk edema	●	●	●	●	●
	Yolk deformation	●	●	●	●	●
	Growth retardation[e]		●	●	●	●
	Hatching			●	●	●
	Swimmbladder development					●
Hepatotoxicity	Yolk absorption			●	●	●
	Liver darkening			●	●	●
Cardiotoxicity	Pericardial edema		●	●	●	●
	Heart morphology			●	●	●
	Heart beating rate (beat/min)				●	●

[a]No clear organs structure is recognized

[b]Malformation of eyes was recorded for the retardation in eye development and abnormality in shape and size.

[c]Presence of none, one or more than two otoliths per sacculus, as well as reduction and enlargement of otic vesicles

[d]Tail malformation was recorded when the tail was bent, twisted or shorter than to control embryos as assessed by optical comparison

[e]Growth retardation was recorded by comparing with the control embryos in a body length (after hatching)

Table S5. Effect of nischarin agonists on viability of the pancreatic cancer cell line panel.

Cell line	***IC50 (µM)**		
	**Rilmenidine**	**Clonidine**	**Moxonidine**
PANC-1	306.8	>1000	>1000
MIA PaCa-2	169.2	>1000	809.4
BxPC-3	367.7	>1000	320.1
Capan-2	>1000	>1000	>1000
HPAF-II	408.9	>1000	#ND
CFPAC-1	579	>1000	ND
SW1990	458.9	>1000	ND
AsPC-1	968.9	ND	ND

*IC50-50% decrease in MTT reduction compared to untreated control; #ND = not determined;
